# Identification and Validation of Genus/Species-Specific Short InDels in Dairy Ruminants

**DOI:** 10.1186/s12917-025-04694-z

**Published:** 2025-03-28

**Authors:** Gianfranco Cosenza, Andrea Fulgione, Sara Albarella, Francesca Ciotola, Vincenzo Peretti, Daniela Gallo, Alfredo Pauciullo

**Affiliations:** 1 Department of Agricultural Science, University of Naples Federico II, Piazza Carlo Di Borbone 1, Portici, 80055 Italy; 2 Department of Veterinary Medicine and Animal Production, University of Naples Federico II, Via Delpino 1, Naples, 80137 Italy; 3 Department of Agricultural, Forest and Food Sciences, University of Turin, Grugliasco, 10095 Italy

**Keywords:** Goat, Sheep, Cattle, Buffalo, Genetic marker, Tetraplex Specific PCR

## Abstract

**Background:**

Over the past thirty years, the identification of species-specific molecular markers has significantly advanced our understanding of genetic diversity in both plants and animals. Among these, short InDels have emerged as vital genomic features, contributing more to sequence divergence than single nucleotide polymorphisms do in closely related species. This study aimed to identify specific InDels for *Bos taurus*, *Bubalus bubalis*, *Capra hircus*, and *Ovis aries* via an in silico approach and validated them in 400 individuals (100 for each species).

**Results:**

We identified and characterized short, specific InDels in the sequences of the *CSN1S1*, *CSN1S2*, *MSTN*, and *PRLR* genes, which can be used for species identification of *Capra hircus, Ovis aries, Bos taurus*, and *Bubalus bubalis*, respectively. We developed a Tetraplex Specific PCR assay to enable efficient discrimination among these species.

**Conclusions:**

This study highlights the utility of InDels as biallelic, codominant markers that are cost-effective and easy to analyse, providing valuable tools for genetic diversity analysis and species identification.

**Supplementary Information:**

The online version contains supplementary material available at 10.1186/s12917-025-04694-z.

## Introduction

In the past three decades, a wide range of molecular markers have been successfully developed for both plants and animals. Among these, the most common include Short Tandem Repeat Polymorphisms (STRP), Variable Number Tandem Repeats (VNTR), Restriction Fragment Length Polymorphism (RFLPs) or Amplified Fragment Length Polymorphisms (AFLPs), Randomly Amplified Polymorphic DNAs (RAPDs) or Inter-simple sequence repeats (ISSRs) and Inter-retrotransposon Amplified Polymorphisms (IRAPs), Allele Specific Associated Primers (ASAPs), Single Nucleotide Polymorphisms (SNPs), and polymorphic INsertions and DELetions (InDels). The last ones are gain or loss of nucleotides in a single *locus* and are the most frequent polymorphisms in mammalian genomes after SNPs [1]. They are classified as short InDels when fewer than 50 nucleotides are involved and long InDels when more than 50 nucleotides are involved [2]. In humans, it is estimated that there is one short InDel every eight nucleotides [1]. However, InDels contribute more to sequence divergence than do SNPs in terms of base differences [2]. With respect to selective pressures on InDels, deletions consistently segregate at lower frequencies than insertions do, both within genes and across the genome. This has been interpreted as evidence of a stronger purifying selection acting on deletions. A possible mechanistic explanation is that deletions have two breakpoints, rather than just one for insertions, making them more likely to disrupt important motifs [2].

Short InDels have been implicated in various genomic evolutionary processes, such as the evolution of genome size, and may play a key role in maintaining optimal intron size [2]. Furthermore, short InDels are more strongly affected by purifying selection and less affected by positive selection than are SNPs. No significant differences have been observed in the impact of balancing or divergent selection between short InDels and SNPs, as both are similarly distributed across the genome and likely respond to indirect selection in the same way. However, a few genomic regions affected by divergent selection were detected by InDels but not by SNPs [3]. In recent years, interest in identifying, mapping, and functionally analysing InDels has increased, as they are useful for studying evolutionary processes, detecting genomic signatures of selection, serving as genetic markers for quantitative trait *loci* (QTL) mapping, for association studies with economically important traits (Marker-Assisted Selection, MAS), and for disease studies [4, 5]. In most cases, these markers are dimorphic intraspecies InDels (Non-Species-Specific indels, NSS) and are mainly used in association studies with traits of interest [6–14]. In contrast, species-specific InDels in ruminants (Target Species-Specific InDels, TSS), have rarely been identified [15], likely due to difficulties in sequence allignment.

This study aimed to (a) identify specific InDels for domestic cattle (*Bos taurus*), river buffalo (*Bubalus bubalis*), goats (*Capra hircus*), and sheep (*Ovis aries*) using an in silico approach and develop polymerase chain reaction (PCR)-based InDel markers; (b) validate the species specificity of the identified InDel markers; and (c) set up a Tetraplex Specific PCR (TetraS-PCR) assay for the discrimination of these four species in biological samples.

## Materials and methods

### Samples

DNA from 400 individuals representing four species from four different genera: *Bubalus, Bos*, *Ovis*, and *Capra* has been analysed. The samples were distributed as follows: 100 samples from *Bubalus bubalis* (Mediterranean Italian River Buffalo breed), 100 from *Bos taurus* (30 Agerolese 20 Piemontese, 25 Podolica, and 25 Bruna cattle), 100 from *Capra hircus* (25 Napoletana, 20 Sarda, 20 Garganica, 20 Alpine, and 15 Maltese goats), and 100 from *Ovis aries* (30 Laticauda, 30 Sarda, and 40 Bagnolese sheep). All the animals were reared in Italy.

The DNA concentration and optical density (260/280 ratio) of each sample were estimated via a Nanodrop ND-2000C Spectrophotometer (Thermo Scientific, Waltham, MA, USA). The A260/A280 ratios for all the DNA samples ranged between 1.8 and 1.96.

### Bioinformatics, sequence analysis and multiple sequence alignment

To identify specific InDels the genomic sequences of all *Artiodactyla* and *Perissodactyla* species available in GenBank were aligned for genes associated with milk and meat traits which are highly conserved across species due to their fundamental role. Homology searches, comparisons of nucleotide sequences among genes, and multiple alignments for InDels discovery were accomplished via NCBI-BLASTN version 2.2.5 [16]. Finally, an extensive literature review was conducted.

### Allele-specific (AS) primer design and single AS‒PCR amplification conditions for InDels identification

αS1-casein (*CSN1S1*), αS2-casein (*CSN1S2*)*,* Myostatin (*MSTN*) and Prolactin receptor (*PRLR*) were selected as target genes for *Capra hircus*, *Ovis aries*, *Bos taurus*, and *Bubalus bubalis,* respectively.

An AS‒PCR mixture with a final volume of 25 μL was prepared for each gene *(MSTN*, *CSN1S1*, and *CSN1S2)* by mixing 100 ng of genomic DNA, 1 × Green GoTaq1 Flexi Buffer, 1.5 mM MgCl_2_, 200 μM each dNTP, 10 pmol of each primer, and 1 U of GoTaq® G2 Flexi DNA Polymerase (Promega–Madison, Fitchburg, WI, USA). The thermal protocol included a first cycle of denaturation at 97 °C for 2 min, annealing at 58 °C (59 °C for the *Ovis aries CSN1S1-*specific amplification) for 45 s and extension at 72 °C for 1 min. This was followed by 35 cycles of denaturation at 97 °C for 45 s, annealing at 58 °C (59 °C) for 45 s, and extension at 72 °C for 1 min. A final cycle was performed at 97 °C for 45 s and 58 °C (59 °C) for 45 and 72 °C for 5 min to complete the reaction.

InDel detection at the *PRLR locus* was carried out according to Cosenza et al*.* [15]. For the other genes, target InDel regions were selected on the basis of the inclusion or exclusion of species-specific short InDels in the reverse primer sequences (Additional file 1).

The primers used were designed via DNASIS-Pro software (Hitachi, Tokyo, Japan) and purchased from Eurofins (Eurofins Genomics, Germany).

Ten percent of the PCR products for each species were Sanger sequenced, for a total of 160 sequences, at CEINGE-Biotecnologie Avanzate (Naples, Italy).

#### TetraS-PCR assay

The primers used for TetraS-PCR were the same as those used for AS-PCR, with the exception of those for *PRLR* amplification in *Bubalus bubalis* and the forward primer used for *CSN1S2* amplification in *Ovis aries*, which were redesigned (Additional file 2).

To set up the TetraS-PCR, three DNA sample combinations were prepared: a) *Bos taurus* and *Bubalus bubalis*, b) *Capra hircus* and *Ovis aries*, and c) a mixture of *Bos taurus*, *Bubalus bubalis*, *Capra hircus* and *Ovis aries*, with equal quantities of reference DNA from each species included in each sample. TetraS-PCR was carried out by adding all four primer pairs (10 pM of each primer) and 100 ng of DNA to the reaction mix (see the previous section).

The thermal profile was the same as that used for AS‒PCR. All the PCR products were analysed directly by electrophoresis on a 3.0% Tris–borate-ethylenediaminetetraacetic acid (TBE) agarose gel (Bio-Rad, Hercules, CA, USA) in 0.5 × TBE buffer and stained with SYBR green nucleic acid stain (Lonza Rockland, Inc., Rockland, ME, USA).

## Results

### In silico species-specific InDels identification

The comparison of available gene sequences in NCBI database for *Artiodactyla* and *Perissodactyla* species led to the identification of specific InDels at the *CSN1S1*, *CSN1S2*, *MSTN* and *PRLR loci* for *Capra hircus*, *Ovis aries*, *Bos taurus*, and *Bubalus bubalis*, respectively.

A comparative analysis of the *CSN1S1* gene sequences available in the databases for different mammals revealed a specific 28 bp insertion (KC951931.1:g.1989–2016insTGTACAATGCCATTAATATATTGTACAA) in the proximal promoter region of this gene in the *genus Capra*. This insertion is localized between two elements of retroposonic origin (full-length Bov-tA elements) [17]. Interestingly, the first 20 nucleotides, which are almost perfectly duplicated, are absent in all species from the *Artiodactyl* and *Perissodactyl* orders (X59856.2:g.9508-9509delTGTACAATGCCATTAATATA). The only exceptions are species that are members of the *genus Ovis* (such as *Ovis aries* and *Ovis ammon*), which instead show a deletion of the second of three repetitions, TGTACAA, found in goats (JN701803.1:g.91–92delTGTACAA) (Additional files 3 and 4).

Similarly, the comparison of all sequences of the *CSN1S2* gene, encoding αs2-casein for major ruminant species available in public databases, revealed a 14 bp deletion in intron 1, which is unique to the *genus Ovis* (KT283354.1:g.643-644delAGAAATCAAATCTT) (Additional file 5). Notably, the absence of this sequence might represent the ancestral condition of the gene, as it is almost conserved in all species belonging to the *Artiodactyla* and *Perissodactyla* orders (Additional file 6). The only exceptions are the species from the *Tilopoda suborder*, which have a deletion in the same region of intron 1 of the *CSN1S2* gene (GenBank nos. OQ730238 and OQ730239) [18].

In the case of the *MSTN* gene, both literature and in silico (GenBank) evaluations have shown that *Bos taurus* is characterized by the deletion of a 16 bp DNA fragment in intron 1 (AB076403.1:g.1207-1208delGAGTAGGTTATGGCTT). In contrast, all species belonging to the *Artiodactyla* and *Perissodactyla* orders, including other species within the *Bos genus* (such as *Bos grunniens, Bos mutus, Bos frontalis, Bos javanicus, Bos gaurus,* and *Bos indicus*), carry the insertion (Additional files 7 and 8).

Finally, a comparison of *PRLR* gene sequences available in the database for the main ruminant species revealed a deletion of the heptamer CACTACC located between nucleotides 1102 and 1103 of exon 10 (3’-UTR) in all species of the *genus Bubalus.* In contrast, this heptamer is highly conserved across all species belonging to the *Artiodactyla* and *Perissodactyla* orders [15] (Additional files 9 and 10). Through genotyping via an AS‒PCR-based method, the authors confirmed the specificity of this genetic marker for bubaline species.

### InDels detection via AS‒PCR

To verify the species specificity of the genetic markers at the *CSN1S1*, *CSN1S2*, and *MSTN loci*, three novel allele-specific PCR methods were developed. Moreover, the method proposed by [15] was adopted for *PRLR*.

To validate and confirm the specific amplification of DNA and the homozygous conditions for each species-specific marker, ten per cent of the PCR products for each species was Sanger sequenced, for a total of 160 sequences, at CEINGE-Biotecnologie Avanzate (Naples, Italy).

### Identification by AS‒PCR of carriers of short InDels at the *CSN1S1* 5’UTR

To identify carriers of the 28 bp insertion (KC951931.1:g.1989–2016insTGTACAATGCCATTAATATATATTGTACAA), the 20 bp deletion (X59856.2:g.9508–9509delTGTACAATGCCATTAATATA), and the 7 bp deletion (JN701803.1:g.91–92delTGTACAA) in the proximal promoter region of the *CSN1S1* gene, AS‒PCR protocols were developed. Using these methods, samples homozygous for the specific InDels in the *CSN1S1* gene were successfully amplified via PCR with only the reverse primers for the 28 bp “insertion”, the 20 bp “deletion”, or the 7 bp “deletion”.

**Fig. 1:**
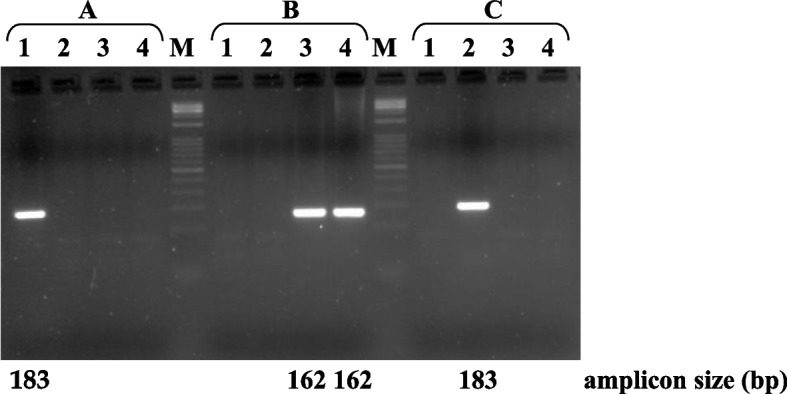
Identification by AS-PCR of carriers of InDels at the *CSN1S1* 5’UTR: (**A**) 28 bp insertion (TGTACAATGCCATTAATATATTGTACAA) (primer reverse: CSN1S1ins28); **B** 20 bp deletion (TGTACAATGCCATTAATATA) (primer reverse: CSN1S1del20), and (**C**) 7 bp deletion (TGTACAA) (primer reverse: CSN1S1del7) in *Capra hircus* (1), *Ovis aries* (2), *Bos taurus* (3), and *Bubalus bubalis* (4). M = Marker 1 kb Opti-DNA Ladder (0.1–10 kb) (Biolabs)

In particular, the AS-PCR reaction mix with the reverse primer specific for the 28 bp insertion (CSN1S1ins28) (Additional file 1) amplified only samples with DNA from *Capra hircus* (183 bp). Conversely, the AS‒PCR mix with the reverse primer for the 20 bp deletion (CSN1S1del20) amplified only DNA from *Bos taurus* and *Bubalus bubalis* (162 bp), whereas the mix with the reverse primer for the 7 bp deletion (CSN1S1del7) amplified only DNA from *Ovis aries* (183 bp) (Fig. 1).

### Identification by AS‒PCR of carriers of the short InDel at *CSN1S2* intron 1

Two allele-specific reverse primers, differing in the presence or absence of the 14 bp sequence (AGAAATCAAATCTT), were designed to verify the species specificity of the genetic marker at intron 1 of the *CSN1S2 locus*.

When the primer pair (F/R) CSN1S2/CSN1S2ins14 was used, only samples homozygous for 14 bp insertion in the *CSN1S2* gene were successfully amplified via PCR (86 bp). In contrast, when the primer pair (F/R) CSN1S2/CSN1S2del14 was used, only samples homozygous for the ‘non-insertion’ allele (76 bp) were successfully amplified.

**Fig. 2:**
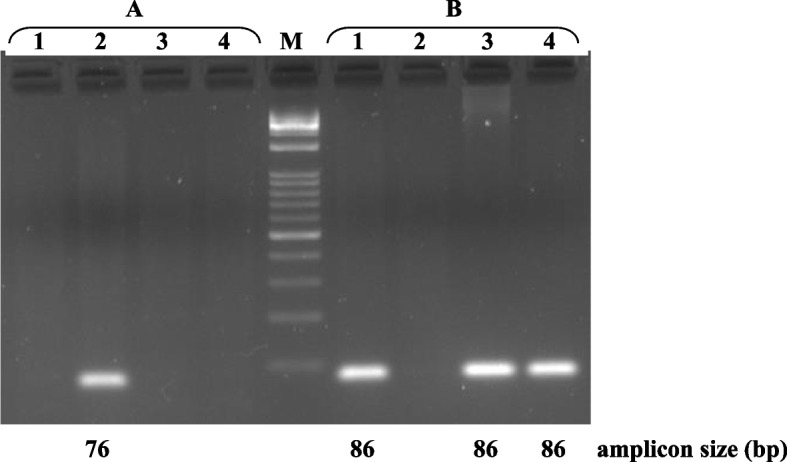
Identification by AS-PCR of carriers of the 14 bp InDel (AGAAATCAAATCTT) at *CSN1S2* intron 1: (**A**) 14 bp deletion (primer reverse: CSN1S2del14); **B** 14 bp insertion (primer reverse: CSN1S2ins14) in *Capra hircus* (1), *Ovis aries* (2), *Bos taurus* (3), and *Bubalus bubalis* (4). M = Marker 1 kb Opti-DNA Ladder (0.1–10 kb) (Biolabs)

Genotyping of the four species from different randomly chosen breeds and genetic types confirmed that only *Ovis aries* samples were characterized by the absence of the 14 bp sequence (Fig. 2).

### Identification by AS‒PCR of carriers of short InDel at *MSTN* intron 1

The species specificity of 16 bp short InDels at intron 1 of the *MSTN* gene was validated via allele-specific primers designed on the basis of the presence or absence of the mutational event.

**Fig. 3:**
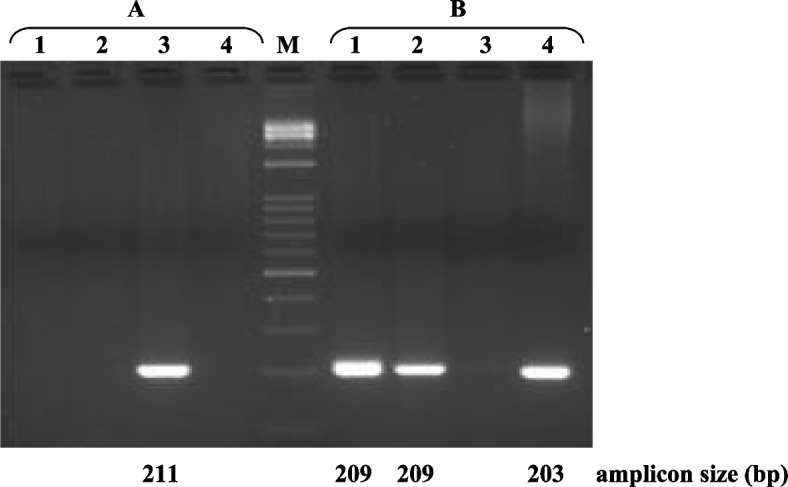
Identification by AS-PCR of carriers of the 16 bp InDel (GAGTAGGTTATGGCTT) at *MSTN* intron 1: (**A**) 16 bp deletion (primer reverse: MSTNdel16); **B** 16 bp insertion (primer reverse: MSTNinsl16) in *Capra hircus* (1), *Ovis aries* (2), *Bos taurus* (3), and *Bubalus bubalis* (4). M = Marker 1 kb Opti-DNA Ladder (0.1–10 kb) (Biolabs)

Specifically, the AS-PCR mixture with the reverse primer MSTNdel16 was successfully amplified exclusively for *Bos taurus* DNA samples (211 bp). Conversely, when the primer specific for the presence of the insertion (MSTNins16) was used, amplification was achieved only for DNA samples from *Bubalus bubalis* (203 bp), *Capra hircus* (209 bp), and *Ovis aries* (209 bp) (Fig. 3).

### Identification by AS‒PCR of InDel carriers at *PRLR* exon 10

To confirm the species specificity of the CACTACC heptamer deletion at exon 10 of the *PRLR* gene for bubaline species, the method proposed by [15] was used.

Genotyping of the 400 DNA samples validated the specificity of this InDel for the bubaline species, thus confirming the findings from the in silico analyses and the literature [15].

### TetraS-PCR assay

To set up the TetraS-PCR assay, new primer pairs (forward and reverse) were designed for the specific amplification of the *PRLR* gene in *Bubalus bubalis*. Additionally, a new forward primer was designed for the specific amplification of *Ovis aries CSN1S2* (Additional file 2).

Initially, simplex PCR analyses of reference DNAs of each species were carried out and revealed that each new primer pair generated species-specific amplification, with no false positives observed in related species. Specifically, the *Bubalus bubalis*-specific primers (PRLRF and PRLRRdel7) (Additional file 2) amplified a 144 bp fragment from *Bubalus bubalis* DNA, with no amplification of *Bos taurus*, *Ovis aries*, or *Capra hircus* DNA. Similarly, the desired amplification (amplicon size of 162 bp) was obtained with only *Ovis aries* DNA when *Ovis aries*-specific primers (CSN1S2F and 3 CSN1S2del14) were used.

For the amplification of *Capra hircus* at the *CSN1S1 locus* and *Bos taurus* at the *MSTN locus*, the primer pairs developed for AS-PCR amplification were used (CSN1S1/CSN1S1ins28–183 bp and MSTN/MSTNdel16–211 bp, respectively) (Additional file 2).

**Fig. 4:**
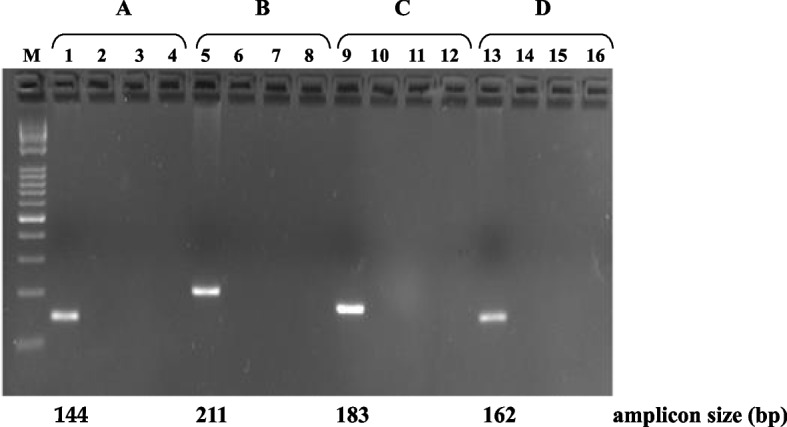
Electrophoretic patterns of simplex species-specific amplification by using new (**A** and **D**) and previously designed (**B** and **D**) specific primer pairs. **A**) *PRLR*; **B**) *MSTN*; **C**) *CSN1S1*; **D**) *CSN1S2*. were from: *Mediterranean river buffalo* (patterns 1, 6, 10 and 14), *Bos taurus* (patterns 2, 5, 11 and 15), *Capra hircus* (patterns 3, 7, 9, 16), and *Ovis aries* (patterns 4, 8, 12 and 13). M: 1 kb Opti-DNA Marker, Applied Biological Materials

Figure 4 shows the electrophoretic patterns of single species-specific amplification via the new primer pairs and the previously designed primers for *CSN1S1* and *MSTN*.

To develop and validate the TetraS-PCR, a mixture of the four primer pairs (Additional file 2) and a mixture of reference DNA from different species were prepared. Briefly, the mixture of the four primer pairs was initially tested on reference DNA from each species and subsequently on 60 DNA mixtures each of two species (30 *Capra hircus*/*Ovis aries* and 30 *Bubalus bubalis*/*Bos taurus*) and 30 DNA mixtures each with all the investigated species.

**Fig. 5:**
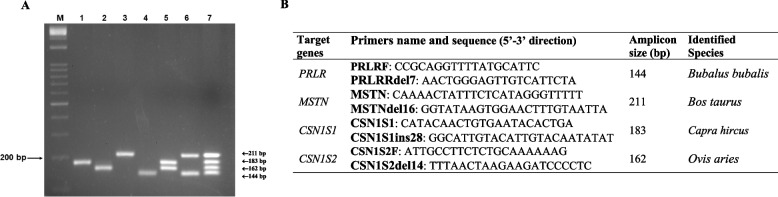
Electrophoretic patterns of amplification products by TetraSS-PCR. Lane 1, *Capra hircus* (183 bp); line 2, *Ovis aries* (162 bp); line 3, *Bos taurus* (211 bp); line 4, Mediterranean river buffalo (144 bp). Lane 5, mixture of DNA from *Capra hircus* and *Ovis aries*; lane 6, mixture of DNA from *Mediterranean river buffalo* and *Bos taurus*; lane 7, DNA amplification products from *Mediterranean river buffalo*, *Bos taurus*, *Capra hircus*, and *Ovis aries*. M) 1 kb Opti-DNA Marker, Applied Biological Materials

The results of the TetraS-PCR assays revealed coamplification of specific fragments in a single PCR, with the sizes of the amplified products being consistent with those of the simplex PCR products. No non-specific bands were observed (Fig. 5).

### Limit of detection (LOD)

The sensitivity of the method for detecting DNA from each species in a mixture was evaluated. Briefly, a mixture of DNA from *Capra hircus*, *Ovis aries*, *Bubalus bubalis*, and *Bos taurus* was serially diluted to the same concentrations (0.1, 0.25, 0.5, 1, 5, 10, and 100 ng/μL) and analysed via multiplex SS‒PCR to determine the sensitivity of the multiplex PCR. The results revealed that the limit of DNA detection was 0.25 ng/μL. The same limit was reported by [19] for the identification of ruminant species in dairy products using PCR assays.

## Discussion

InDels, like other genetic markers such as SNPs, are biallelic, codominant, abundant, and randomly distributed across genomes. When located in functional sequences, InDels are generally subject to stronger purifying selection than are SNPs. Long InDels and those affecting multiple functionally constrained nucleotides undergo stronger purifying selection [1]. One key advantage of InDels over SNPs is their lower likelihood of multiple mutations of the same length occurring at the same genomic position, making them useful for studying genetic relationships. Shared InDels represent identity by descent [20] and can serve as ancestry-informative markers, aiding in the study of population substructure, genetic diversity, phylogenetic relationships, genome evolution, and functional divergence [21].

Compared with SNPs, InDels contribute more significantly to sequence divergence, especially among closely related species [22, 23]. They are useful markers for population genetics, taxon diagnoses, forensic genetics, genetic mapping, association studies, and species identification [24–27].

Despite the advantages and broad applications of these markers, InDel studies remain underrepresented in the literature. Specifically, to our knowledge, no short genus- or species-specific short InDels have been identified in livestock ruminants to date. The only exception is the *PRLR* gene, which encodes the prolactin receptor, a member of the growth hormone/prolactin receptor gene family involved in various endocrine and reproductive functions [15].

The *PRLR* gene has been mapped to chromosome 19 in *Bubalus bubalis* (NC_059175.1 from 38,740,723 to 38,937,314), chromosome 20 in *Bos taurus* (NC_037347.1 from 38,915,987 to 39,108,971) and *Capra hircus* (NC_030827.1 from 38,891,738 to 39,090,307), and chromosome 16 in *Ovis aries* (NC_056069.1 from 39,248,092 to 39,250,921). It consists of 10 exons, with exons 1 and 2 being non-coding [15, 28, 29]. Two distinct *PRLR* isoforms are produced through alternative splicing of exon 10 of the primary transcript.

This last DNA coding region has been observed to accumulate a significant number of inter- and intraspecies polymorphisms across the different species investigated. Notably, a deletion of a CACTACC heptamer between nucleotides 1102 and 1103 of exon 10 has been identified as a *genus*-specific DNA marker for the *genus Bubalus* [15].

Since no short InDels specific to other widely distributed ruminant genera, such as *Bos*, *Ovis*, and *Capra,* have been reported, we conducted an extensive bibliographic review and comparative analysis of gene sequences from various mammalian species, focusing on key traits such as meat and milk production, which are highly conserved across species due to their fundamental role.

For instance, milk and mammary genes are highly conserved across all mammals, evolving more slowly along the lineage and subject to stronger selective constraints than most other genes in the genome. These genes have been shown to experience significant negative selection compared with the rest of the genome [30].

Among them, genes encoding the αs1 and αs2 casein fractions (*CSN1S1* and *CSN1S2*, respectively) are known to influence both the qualitative and quantitative properties of milk in major ruminant and non-ruminant species of zootechnical interest. Due to their functional importance, structure, and variability, these genes serve as powerful molecular models for evolutionary research. They also provide insights into the genetic architecture of lesser-studied species and the phylogenetic relationships among mammalian species and domestic animals [18, 31–36]. Both genes have been annotated in nearly all species (https://www.ncbi.nlm.nih.gov/gene) and are particularly well characterized in *Capra hircus*, *Bos taurus*, *Ovis aries* and *Bubalus bubalis*. These genes share a similar organization among ruminants, although there are differences in intron size, mainly due to the varying distributions of artiodactyl retroposons located in introns and regulatory regions [17, 31, 32, 36–41]. The *CSN1S1* and *CSN1S2* genes are clustered with the β and k casein encoding genes (*CSN2* and *CSN3*, respectively) in a 250 kb (kilobase) region on chromosome 6 in *Capra hircus*, *Ovis aries* and *Bos taurus* and on chromosome 7 in *Bubalus bubalis* [32]. An exception to this organization is found in certain mammals, such as donkeys, horses, rabbits, and rodents, which have an extra copy of the *CSN1S2* gene, indicative of a recent paralogous gene duplication event [34, 42, 43].

The *MSTN* gene encodes myostatin, also known as growth differentiation factor 8 (GDF8), a negative regulator of skeletal muscle growth and size; therefore, its role in meat production is highly interesting. Mutations at this *locus* result in muscular hypertrophy and reduced fat, a phenomenon observed in ‘double muscled’ breeds of cattle and in several livestock and model species [44, 45]. The *MSTN* gene has been mapped to chromosome 2 in *Bos taurus* (NC_037329.1 from 6,278,864 to 6,285,491), *Capra hircus* (NC_030809.1 from 130,227,819 to 130,232,923, complement*), Bubalus bubalis* (NC_059158.1 from 58,423,897 to 58,428,995), and *Ovis aries* (NC_056055.1 from 119,285,858 to 119,292,614). This gene consists of three exons and two introns, and its sequence has been highly conserved across vertebrate species throughout evolution [46–48].

The deletion of a 16 bp DNA fragment at intron 1 of the *MSTN* gene identified in this study appears to be specific to *Bos taurus* compared to other species within the *Bos* genus (*Bos grunniens*, *mutus*, *gaurus*, *frontalis* and *javanicus*), as well as to species in the genera *Bubalus*, *Capra*, and *Ovis* (Additional file 7). In some *Bos indicus* breeds, such as Guanling, Weizhou, Weining, Lincanggaofeng, Brahman (an American zebuine × taurine hybrid beef cattle breed), and Qinchuan cattle [49, 50], comparative analysis revealed sequences with and without mutations from data available in GenBank. Furthermore, for the indicine Nelore and Gir cattle breeds, only one genomic sequence is available, and both lack the insertion. A single sequence containing the 16 bp DNA insertion is reported for Yunling cattle (Additional file 7).

All these variations could be due to the hybrid origin of these breeds [51–60]. Cattle are generally categorized into two major types: zebu (humped) and taurine (without humps), which are often classified as distinct species (*Bos taurus indicus* and *Bos taurus taurus*). However, due to their complete interfertility, they are frequently considered subspecies [61]. Genetic introgression (from *Bos indicus* to *Bos taurus*, but not the reverse) has been widely used to create new composite cattle populations, combining the climatic resilience of *Bos taurus indicus* with the relatively high dairy and meat productivity of *Bos taurus taurus* [62, 63].

For example, Chinese cattle are categorized into three groups on the basis of geographic distribution: southern, central and northern. The southern and northern groups primarily descend from *Bos indicus* and *Bos taurus*, respectively, whereas the central group originates from both [54]. Similarly, in Brazil, one of the world’s leading beef exporters, nearly 80% of the cattle herd is believed to have some degree of *Bos indicus* influence [64].

To further support the specificity of the InDel at the *MSTN locus*, a study on 722 *Bos taurus* cattle from ten different breeds (Hereford, Angus, South Devon, Composite, Charolais, Red Poll, Shorthorn, Simmental, Murray Grey) and cross-bred (Holstein–Friesian x Jersey) did not detect the insertion [65]. However, it was observed exclusively in the homozygous state by [66] in four different breeds of *Bos indicus* raised in India (Rathi, Deoni, Idduki and Vatakara).

Based on the findings from this study, the available data in GenBank, and the literature, the short InDels identified and characterized at the *MSTN locus* serve as effective markers for discriminating between *Bos taurus* and other species within the *Bos genus*.

In contrast, it is possible that the short InDels detected at the *PRLR*, *CSN1S1*, and *CSN1S2 loci* can serve as valid markers for discriminating between the *genera Bubalus*, *Capra*, and *Ovis*, respectively (Additional files 4, 5, and 10).

Among the various potential applications of genus- or species-specific InDels, one of the most practical is the development of protocols for the traceability of animal-derived products. Food adulteration, involving the admixture or substitution of high-value species-specific ingredients with less expensive, lower-quality ingredients, is an increasingly common issue. The inclusion of undeclared species in food ingredients can also trigger allergic reactions, posing a significant health risk to consumers [67, 68].

Currently, several analytical methods have been developed for food traceability [69, 70], but molecular analyses (DNA-based assays), due to their sensitivity, accuracy, and reproducibility, offer particularly valid tools [70, 71].

Only a few examples in the literature report the use of short and long interspersed repetitive elements as markers for DNA-based species identification [19, 72].

The genotyping of short InDel markers has several advantages. They are easier to analyse than SNPs, require less expensive procedures, and rely on equipment readly available in most laboratories [70].

Moreover, InDel markers are well suited for establishing protocols to identify hybrids through direct size separation of DNA fragments and are effective in amplifying and typing mixed or highly degraded DNA samples.

To identify genus/species-specific short Indels for ruminants, a TetraS-PCR assay was developed, employing four primer pairs, each designed to target distinct genes within a single reaction. A critical aspect of the setup of the TetraS-PCR system is the design of primers tailored to be species specific.

An undeniable advantage of this approach is the higher annealing efficiency of primers, stemming from their design based on species-specific InDel sequences. The selection of target regions containing InDels followed two main criteria: (a) short amplicon size to overcome amplification issues due to fragmented DNA in processed products and (b) significant yet comparable size differences among species-specific amplicons to allow clear and unambiguous separation via electrophoresis.

The proposed method, which is based on the detection of short InDels, allows for the simultaneous discrimination of *Bubalus bubalis*, *Bos taurus*, *Capra hircus*, and *Ovis aries* in animal-derived products or forensic samples.

Specifically, this method could be useful for discriminating between taurine and indicine meat, as well as identifying meats derived from various genetic combinations. *Bos indicus* beef is generally leaner, tougher, and of lower quality than *Bos taurus* [73]. As a result, some labelling systems exclude zebu meat from their certified brands, making robust species discrimination methods essential for maintaining consumer trust and product authenticity. Consequently, due to the increasing meat imports from countries where crossbreeding is widely practiced, it becomes crucial to have adequate tools to ensure the origin and quality of the meat.

Additionally, the proposed method can be a useful tool for detecting the adulteration of sheep milk with goat milk or the inclusion of cow milk in both. Fraudulent practices such as the adulteration of goat and sheep milk with cow milk are common. According to EU Directive No. 273/2008, the addition of cow milk to goat and sheep milk products is regulated, with a permissible limit set at 0.5%. Beyond the economic implications stemming from the high cost of sheep and goat milk, the primary risk for consumers lies in the unintentional consumption of proteins with high allergenic potential, such as bovine αs1 casein and β-lactoglobulin [74].

The relatively high cost and seasonal availability of buffalo milk are the main factors responsible for the well-known fraud related to “Mozzarella di Bufala Campana”, a typical Italian product certified by the European Protected Designation of Origin (PDO) (EC Regulation No. 1107/96 of 12 June 1996). Buffalo milk is characterized by a higher fat content (on average 8.0%) and casein concentration (averaging 36.7 mg/ml) than milk from other ruminants [75, 76]. Since the composition of milk from different species significantly influences cheese yield, flavour and sensory properties, adulterating the milk used in mozzarella production compromises the quality and sensory attributes of the final product, resulting in outcomes that are below consumer expectations.

Other potential applications of the proposed method include the detection of ruminant milk components in commercial milk from minor dairy species, such as equids (donkeys or horses) or camelids (dromedary and bactrian camels). These species produce milk that is nutritionally beneficial for human health, leading to a growing international interest in their health-promoting properties, including antibacterial and antiviral effects, as well as the diverse range of foods derived from their milk [34, 77]. As with the examples previously mentioned, the adulteration of such milk is often economically motivated, driven primarily by low milk production and the significant price difference between their milk and that of more common species, which may have a similar appearance and taste [78, 79].

The need for robust and reliable methods in animal forensic genetics is becoming increasingly critical due to the growing role of non-human DNA evidence in investigative contexts. The use of InDel markers can significantly contribute to forensic scenarios requiring species identification, including linking suspects, victims, and crime scenes; investigating animal attack; protecting wildlife; and combating illegal trade [80]. Moreover, differentiating between closely related species, such as *Bos* and *Bubalus*, especially in degraded samples, is also essential for advancing zooarchaeological studies [81].

In conclusion, this study provides new insights into the genetic differentiation among diverse group combinations, an area that remains underexplored. Finally, for the first time, a single genetic marker was identified and characterized, enabling the reliable distinction between taurine and zebu cattle at the DNA level.

## Supplementary Information

Additional file 1: Table 1: Gene target, primer sequence, amplified species and amplicon size of AS-PCRs.

Additional file 2: Table 2: Gene target, primer sequence and amplicon size of Tetraplex Specie-Specific PCR.

Additional file 3: Fig. 1: Comparison of the partial proximal *CSN1S1* promoter sequences of representative species from the *Artiodactyla* and *Perissodactyla* orders. The dashes represent nucleotides identical to those in the upper lines. The short InDels are highlighted in gray, TGTACAA repetitions are in bold, and duplications are underlined. 1: *Capra hircus* (GenBank KC951931.1 from 1943 to 2062); *Capra ibex* (GenBank SJYO01041852.1 from 71532893 to 71533011); *Capra aegagrus* (GenBank CBYH010035613.1 from 223376 to 223494); *Capra falconeri* (GenBank JAWPPH010101386.1 from 2986 to 3104, complement); 2: *Ovis aries* (GenBank JN701803.1 from 25 to 136); *Ovis ammon* (GenBank NIWH01032118.1 from 68020430 to 68020541); 3: *Bubalus bubalis* breed Mediterranean (GenBank AWWX01570220.1 from 20546 to 20642), *Bubalus bubalis* breed Murrah (GenBank AF529305.2 from 1027 to 1124), *Bubalus kerabau* breed swamp (GenBank JARFXY010000007.1 from 89615493 to 89615589), *Bubalus depressicornis* (GenBank JAMXBS010096084.1 from 20891 to 20987), and *Bos grunniens* (GenBank VBZB01000005.1 from 35968966 to 35969062); 4: *Bos taurus* (GenBank X59856.2 from 9464 to 9561), and *Bos indicus* (GenBank JASFDU010376321.1 from 1210 to 1307); 5: *Rangifer tarandus *(GenBank OX596114.1 from 19755123 to 19755217); 6: *Cervus elaphus *(GenBank OU343083.1 from 30458569 to 30458663); 7: *Camelus dromedarius* (GenBank LSZX01093722.1 from 59067 to 59161), and *Camelus ferus* (GenBank AGVR01039100.1 from 241242 to 241336); 8: *Lama glama* (GenBank PNXU01093137.1 from 152525 to 152619), and *Vicugna pacos* (GenBan ABRR03000026.1 from 54448393 to 54448487); 9: *Sus scrofa* (GenBank EU025875.1 6815 to 6915);10: *Ceratotherium simum* (GenBank PVLE01002027.1 from 8152 to 8251); 11:*Tapirus indicus* (GenBank PVIE01006105.1 from 63615 to 63715).

Additional file 4: Table 3 *In silico* species-specific InDel identification at the *CSN1S1 locus* in ruminant species. The specific insertion for the *genus Capra* is highlighted in gray, and the deletion specific for the *genus Ovis *is in bold.

Additional file 5: Table 4 *In silico* species-specific InDel identification at the *CSN1S2 locus* in *the genera Capra*, *Ovis*, *Bubalus*, and *Bos*. The specific deletion for the *genus* *Ovis* is highlighted in gray.

Additional file 6: Fig. 2 Comparison of *CSN1S2* partial intron 1 nucleotide sequences of representative species belonging to the *Artiodactyla* and *Perissodactyla* orders. The dashes represent nucleotides identical to those in the upper lines. The short InDels are highlighted in gray. 1: *Ovis aries* (GenBank KT283354.1 from 610 to 696); 2: *Ovis aries* (GenBank JAWMPZ010000006.1 from 91390711 to 91390797); 3: *Capra hircus* (GenBank LWLT01000006.1 from 86078347 to 86078447); 4: *Bubalus bubalis* (Mediterranean GenBank MW159135.1 from 2404 to 2504, and Murrah GenBank VDCC01000007.1 from 32346792 to 32346892); 5: *Bubalus bubalis* (Mediterranean breed GenBank MW159136.1 from 2393 to 2493); 6: *Bubalus bubalis* (Kerabau swamp GenBank JARFXY010000007.1 from 89744003 to 89744103, and *Depressicornis* GenBank JAMXBS010059713.1 from 4606 to 4706); 7: *Bos taurus* (GenBank M94327.1 from 3584 to 3684); 8: *Bos indicus *(GenBank PRDE01000026.1 from 87254016 to 87254116); 9: *Bos grunniens* (GenBank VBZB01000005.1 from 35840127 to 35840227); 10: *Rangifer tarandus* (GenBank OX596114.1 from 19874491 to 19874593), and *Cervus elaphus* (GenBank OU343083.1 from 30339433 to 30339535, complement); 11: *Muntiacus reevesi* (GenBank OZ005646.1 from 30071163 to 30071265); 12: *Sus scrofa* (GenBank LUXU01069711.1 from 564154 to 564256); 13: *Equus caballus* (GenBank JAPJZS010003086.1 from 51148228 to 51148330), *Equus asinus* (GenBank JREZ01000259.1 from 17762 to 17864), and *Equus quagga* (GenBank JAKJSB010001568.1 from 100903916 to 100904018, complement); 14: *Ceratotherium simum* (GenBank AKZM01002854.1 from 54353 to 54455); 15: *Tapirus indicus* (GenBank JAVSPQ010000004.1 from 54488574 to 54488676).

Additional file 7: Table 5 *In silico* species-specific InDel identification at the *MSTN locus* in *the genera Capra*, *Ovis*, *Bubalus*, and *Bos*. The specific deletion for *Bos taurus* is highlighted in gray.

Additional file 8: Fig. 3 Comparison of the partial *MSTN* intron 1 nucleotide sequences of representative species belonging to the *Artiodactyla* and *Perissodactyla* orders. The dashes represent nucleotides identical to those in the upper lines. The short InDels are highlighted in gray. 1: *Bos taurus* (GenBank AB076403.1 from 1159 to 1262), and *Bos indicus* x *Bos taurus* hybrids (GenBank PUFT02000002.1 from 127935602 to 127935705); 2: *Bos grunniens* (GenBank JN642607.1 from 1097 to 1216), *Bos mutus* (GenBank VBQZ03000004.1 from 47972658 to 47972777), *Bos frontalis *(GenBank RBVW01003794.1 from 79189 to 79308), *Bos javanicus* (GenBank JAVLEU010000002.1 from 6312509 to 6312628), *Bos gaurus* (GenBank JACAOC010000017.1 from 1124918 to 1125037), *Bos indicus* (GenBank AY794986.1 from 1159 to 1278), *Bos indicus* x *Bos taurus *hybrids (GenBank PUFS02000002.1 from 6036628 to 6036747), *Bison bison* (GenBank JPYT01086742.1 from 5189 to 5308), and *Bos taurus* breed Yunling (GenBank JAWKDW010000003.1 from 131646859 to 131646740, complement); 3: *Bubalus kerabau* breed swamp (GenBank JARFXY010000003.1 from 59857473 to 59857592), and *Bubalus depressicornis* (GenBank JAMXBS010027670.1 from 21336 to 21455); 4: *Bubalus bubalis* breed Mediterranean (GenBank DQ091762.1 from 1065 to 1184), and *Bubalus bubalis* breed Murrah (GenBank VDCC01000002.1 from 58035442 to 58035561); 5: *Rangifer tarandus* (GenBank OX596096.1 from 68947317 to 68947436), and *Cervus elaphus* (GenBank OU343110.1 from 10575652 to 10575771); 6: *Ovis aries* (GenBank MH025940.1 from 2210 to 2329), and *Capra hircus* (GenBank JX078969.1 from 1224 to 1343); 7: *Camelus dromedarius* (GeneBan LSZX01094446.1 from 13519 to 13638), *Camelus ferus* (GeneBanVSZR01000033.1  from 59262525 to 59262644), and *Camelus bactrianus* (GenBank CAOW010387223.1 from 1829 to 1948); 8: *Lama glama* (GeneBan PNXU01000637.1 from 236134 to 236253), and *Vicugna pacos* (GenBank JEMW01017296.1 from 84546 to 84665); 9: *Sus scrofa *(GenBank EF490990.1 from 2235 to 2353); 10: *Equus caballus* (GenBank KY746357.1 from 1645 to 1763); 11: *Equus asinus* (GenBank JADWZW020000004.1 from 25419042 to 25419156); 12: *Equus quagga* (GenBank JAKJSB010006700.1 from 8862462 to 8862576, complement); 13: *Ceratotherium simum* (GenBank PVLE01086917.1 from 2942 to 3062); 14: *Tapirus indicus* (GenBank PVIE01000017.1 from 143272 to 143391).

Additional file 9: Fig. 4 Comparison of the *PRLR* sequences of partial exon 10 with the homologous sequences available in the database for the main species belonging to the *Artiodactyla* and* Perissodactyla* orders. The dashes represent nucleotides identical to those in the upper lines. The short Indels are highlighted in gray. 1: *Bubalus bubalis *(Mediterranean, GenBank MF461277.1 from 12141 to 12193, Murrah GenBank XM_025270632.3 from 2207 to 2259, Kerabau swamp, GenBank JARFXY010000018.1 from 40954223 to 40954275, Depressicornis, GenBank JAMXBS010060037.1 from 50052 to 50104, and *Syncerus caffer*, GenBank SJXX02054938.1 from 1119837 to 1119889, complement); 2: *Bos taurus* (GenBank OY997248.1 from 42476450 to 42476509), *Bos indicus* (GenBank XM_019983032.1 from 2188 to 2247), *Bos javanicus* (GenBank XM_061393756.1 from 2862 to 2921), *Bos mutus* (GenBank XM_005907371.2 from 1947 to 2006), and *Bison bison* (GenBank XM_010853807.1 from 2122 to 2181); 3: Tragelaphus oryx (GenBank SJYK013989325.1 from 1715 to 1774, complement); 4: *Capra hircus* (GenBank XM_061393756.1 from 2759 to 2818), and *Ovis aries* (GenBank KC734660.1 from 503 to 562); 5: *Cervus elaphus* (GenBank XM_043887317.1 from 2217 to 2278), and *Rangifer tarandus* (GenBank OX459950.2 from 41714039 to 41714100); 6: *Camelus dromedarius* (GenBank XM_031443888.1 from 2286 to 2349), *Camelus ferus* (GenBank XM_006194514.3 from 2238 to 2301), and *Camelus bactrianus* (GenBank XM_010955538.2 from 2224 to 2287); 7: *Vicugna pacos* (GenBank XM_006199659.3 from 2235 to 2298); 8: *Lama glama* (GenBank DQ206831 from 1068 to 1127); 9: *Sus scrofa *(GenBank HM059030.1 from 1105 to 1169); 10: *Equus asinus* (GenBank XM_014831229.2 from 2492 to 2549), and *Equus quagga *(GenBank XM_046671632.1 from 2414 to 2477; 11: *Equus caballus* (GenBank XM_001500104.4 from 2757 to 2820); 12: *Ceratotherium simum simum* (GenBank XM_014781582.1 from 2216 to 2279); 13: *Tapirus indicus* (GenBank JAVSPQ010000006.1 from 118655899 to 118655962).

Additional file 10: Table 6: *In silico* species-specific InDel identification at the *PRLR locus* in *the genera Capra*, *Ovis*, *Bubalus*, and *Bos*. The specific deletion for the *genus* *Bubalus *is highlighted in gray.

Additional file 11: Uncropped gels used for Figure 1, 2, 3, 4 and 5.

## Data Availability

In this study no new DNA sequences and polymorphisms have been generated. The datasets used and analysed for in silico analyses were retrieved from the public repository https://www.ncbi.nlm.nih.gov/nuccore/?term= . The authors are available for any clarifications on the protocols developed.

## Abbreviations

AS‒PCR: Allele-specific PCR
*CSN1S1*: αS1-casein (gene)
*CSN1S2*: αS2-casein (gene)
*CSN2*: β Casein (gene)
InDels: INsertions and DELetions
*MSTN*: Myostatin (gene)
PCR: Polymerase chain reaction
*PRLR*: Prolactin receptor (gene)
SNPs: Single Nucleotide Polymorphisms
TetraS-PCR: Tetraplex-Specific PCR
TSS: Target Species-Specific
