# Supplementary material for: Identification and Validation of Genus/Species-Specific Short InDels in Dairy Ruminants
Source: BMC Vet Res. 2025 Mar 28;21:215. doi: 10.1186/s12917-025-04694-z (PMC11951546; doi:10.1186/s12917-025-04694-z)
Supplement: Supplementary file 1 — Additional file 1: Table 1: Gene target, primer sequence, amplified species and amplicon size of AS-PCRs. [file 12917_2025_4694_MOESM1_ESM.pdf]

Additional File 1 - Table 1 Gene target, primer sequence, amplified species and amplicon size of AS-PCRs.

**Target genes**	**Primers name and sequence (5’-3’ direction)**		**Amplified species and amplicon size (bp)**	**Amplified DNA region**	**Ta (°C)**
[1]*CSN1S1*	Forward common primer	CSN1S1: CATACAACTGTGAATACACTGA		5′ flanking region	
	Allele-specific reverse primer	CSN1S1ins28: GGCATTGTACATTGTACAATATAT	*Capra hircus* (183)		58
	Allele-specific reverse primer	CSN1S1del20: ATTAACTGGCATTGTACA-------ATATATT	*Ovis aries* (183)		59
	Allele-specific reverse primer	CSN1S1del7: GGCATTGTACATTGTACAA--------------------AAATAT	*Bos taurus* (162), *Bubalus bubalis* (162)		58
[2]*CSN1S2*	Forward common primer	CSN1S2: GTAGTCAGCAAACCTTGG		Partial intron 1	58
	Allele-specific reverse primer	CSN1S2del14: TTTAACTAAGAAGATCCCCTC	*Ovis aries* (76)		
	Allele-specific reverse primer	CSN1S2ins14: ACTAAGAAGATTTGATTTCTAAGATC	*Bos taurus* (86), *Capra hircus* (86), *Bubalus bubalis* (86)		
[3]*MSTN*	Forward common primer	MSTN: CAAAACTATTTCTCATAGGGTTTTT		Partial intron 1	58
	Allele-specific reverse primer	MSTNdel16: GGTATAAGTGGAACTTTGTAATTA	*Bos taurus* (211)		
	Allele-specific reverse primer	MSTNins16: TAAGCCATAACCTACTCAATTA	*Bubalus bubalis* (203), *Capra hircus* (209), *Ovis aries* (209)		
[4]*PRLR*	Forward common primer	PRLR: CTCTGCTAAACCCTTGGAAT		Partial exon 10	60
	Allele-specific reverse primer	PRLRdel7: AACTGGGAGTTGTCATTCTAA	*Bubalus bubalis* (455)		
	Allele-specific reverse primer	PRLRins7: AACTGGGAGTTGGGTAGTG	*Bos taurus* (462), *Capra hircus* (462), *Ovis aries* (462)		

1. Primers were designed according to goat sequence (GenBank accession n. KC951931.1) and by comparison with the corresponding cattle, sheep and buffalo sequences (GenBank accession nos. X59856.2 1, JN701803.1, and AF529305.2, respectively).
2. Primers were designed according to sheep sequence (GenBank accession n. KT283354.1) and by comparison with the corresponding cattle, buffalo, and goat sequences (GenBank accession nos. X59856.2, AF529305.2, and LWLT01000006.1, respectively).
3. Primers were designed according to cattle sequence (GenBank accession n. AB076403.1) and by comparison with the corresponding buffalo, sheep, and goat sequences (GenBank accession nos. DQ091762.1, MH025940.1, and JX078969.1, respectively).
4. According to Cosenza *et al*. (2018)

All allele-specific reverse primers were designed on the sequences of the 4 investigated species assuming the absence/presence of the species-specific InDels (highlighted in grey)
