# Supplementary material for: Identification and Validation of Genus/Species-Specific Short InDels in Dairy Ruminants
Source: BMC Vet Res. 2025 Mar 28;21:215. doi: 10.1186/s12917-025-04694-z (PMC11951546; doi:10.1186/s12917-025-04694-z)
Supplement: Supplementary file 2 — Additional file 2: Table 2: Gene target, primer sequence and amplicon size of Tetraplex Specie-Specific PCR. [file 12917_2025_4694_MOESM2_ESM.pdf]

Additionally file 2 - Table 2 Gene target, primer sequence and amplicon size of Tetraplex Specie-Specific PCR.

**Amplified Species**	**Target genes**	**Accession number**	**Primers name and sequence (5'-3' direction)**	**Nucleotides position (from – to)**	**Amplified DNA region**	**Amplicon size (bp)**	**TM (°C)**
Bubalus bubalis	*PRLR*	MF461277.1	**PRLRF**: CCGCAGGTTTTATGCATTC<br>**PRLRRdel7**: AACTGGGAGTTGTCATTCTA	12031-12049<br>Complementary to nt 12155-12174	Partial exon 10	144	58
Bos taurus	*MSTN*	JQ711180.1	**MSTN**: CAAAACTATTTCTCATAGGGTTTTT<br>**MSTNdel16**: GGTATAAGTGGAACTTTGTAATTA	1608-1632<br>Complementary to nt 1800-1818	Partial intron 1	211	
Capra hircus	*CSN1S1*	AJ504710.2	**CSN1S1**: CATACAACTGTGAATACACTGA<br>**CSN1S1ins28**: GGCATTGTACATTGTACAATATAT	813-834<br>Complementary to nt 972-995	5′ flanking region	183	
Ovis aries	*CSN1S2*	KT283352.1	**CSN1S2F**: ATTGCCTTCTCTGCAAAAAAG<br>**CSN1S2del14**: TTTAACTAAGAAGATCCCCTC	492-512<br>Complementary to nt 633-653	5′ flanking region	162	
