# Supplementary material for: Identification and Validation of Genus/Species-Specific Short InDels in Dairy Ruminants
Source: BMC Vet Res. 2025 Mar 28;21:215. doi: 10.1186/s12917-025-04694-z (PMC11951546; doi:10.1186/s12917-025-04694-z)
Supplement: Supplementary file 3 — Additional file 3: Fig. 1: Comparison of the partial proximal CSN1S1 promoter sequences of representative species from the Artiodactyla and Perissodactyla orders. The dashes represent nucleotides identical to those in the upper lines. The short InDels are highlighted in gray, TGTACAA repetitions are in bold, and duplications are underlined. 1: Capra hircus (GenBank KC951931.1 from 1943 to 2062); Capra ibex (GenBank SJYO01041852.1 from 71532893 to 71533011); Capra aegagrus (GenBank CBYH010035613.1 from 223376 to 223494); Capra falconeri (GenBank JAWPPH010101386.1 from 2986 to 3104, complement); 2: Ovis aries (GenBank JN701803.1 from 25 to 136); Ovis ammon (GenBank NIWH01032118.1 from 68020430 to 68020541); 3: Bubalus bubalis breed Mediterranean (GenBank AWWX01570220.1 from 20546 to 20642), Bubalus bubalis breed Murrah (GenBank AF529305.2 from 1027 to 1124), Bubalus kerabau breed swamp (GenBank JARFXY010000007.1 from 89615493 to 89615589), Bubalus depressicornis (GenBank JAMXBS010096084.1 from 20891 to 20987), and Bos grunniens (GenBank VBZB01000005.1 from 35968966 to 35969062); 4: Bos taurus (GenBank X59856.2 from 9464 to 9561), and Bos indicus (GenBank JASFDU010376321.1 from 1210 to 1307); 5: Rangifer tarandus (GenBank OX596114.1 from 19755123 to 19755217); 6: Cervus elaphus (GenBank OU343083.1 from 30458569 to 30458663); 7: Camelus dromedarius (GenBank LSZX01093722.1 from 59067 to 59161), and Camelus ferus (GenBank AGVR01039100.1 from 241242 to 241336); 8: Lama glama (GenBank PNXU01093137.1 from 152525 to 152619), and Vicugna pacos (GenBan ABRR03000026.1 from 54448393 to 54448487); 9: Sus scrofa (GenBank EU025875.1 6815 to 6915);10: Ceratotherium simum (GenBank PVLE01002027.1 from 8152 to 8251); 11:Tapirus indicus (GenBank PVIE01006105.1 from 63615 to 63715). [file 12917_2025_4694_MOESM3_ESM.pdf]

```
TTAAAGCCCTCACT TGTATAGATATTTTATTTAGCACATAATATTTTGTACAATGCCATTAATATATTGTACAATGTACAATGCCAGTTAATTCTAGGAGTACAATTAAGAATTGGAGG 1
-------------- ----C------------------------------------------------       -----------------------------C--------------- 2
-------------- -------------- -----------------                    --C-------------------------------------------------A 3
-------T------ ---------T-A-- -----------------                    --C-------------------------------------------------A 4
C----A-------- -----A    ----------------------                    --C--A---A----------------------------G-------C-----A 5
-----A-------- ------    ----------------------                    --C--A----------------------------------------C-----A 6
G---- AA---TAT  ----G--------C-C-----T------G---                      -TA-G----TT-------------------------------T------A 7
G---- AA---TA-  ----G--------C-------T------G---                      -TA-G----TT-------------------------------T------A 8
-----T--AG-T--A-----------------C----TGC---T----                   -CC--A---TA----------------------T--T-T--C--G-C----AA 9
-A---A-----TTCA--C--G--------C--C---GT----------                    -C-------------------A-------------G----G--T-------A 10
-AG--T-----T-CA--C--G--------C--C-T--T--G-------                    -C-------------------A-------------G----G--G-------A 11
```

Sequences	SUBORDERS	ORDERS
1–6	Ruminantia	Artiodactyla
7–8	Tylopoda	Artiodactyla
9	Suiformes	Artiodactyla
10–11	Ceratomorpha	Perissodactyla
