# Supplementary material for: Identification and Validation of Genus/Species-Specific Short InDels in Dairy Ruminants
Source: BMC Vet Res. 2025 Mar 28;21:215. doi: 10.1186/s12917-025-04694-z (PMC11951546; doi:10.1186/s12917-025-04694-z)
Supplement: Supplementary file 4 — Additional file 4: Table 3 In silico species-specific InDel identification at the CSN1S1 locus in ruminant species. The specific insertion for the genus Capra is highlighted in gray, and the deletion specific for the genus Ovis is in bold. [file 12917_2025_4694_MOESM4_ESM.pdf]

Additional file 4 - Table 3. *In silico* species-specific InDel identification at *CSN1S1 locus* in ruminant species. The specific insertion for the *genus Capra* is highlighted in grey, and the deletion specific for the *genus Ovis* is in bold.

Gene	Genus	Species/ hybrid	GeneBank	InDels
CSN1S1	*Capra*	*Capra hircus*	KC951931.1; AJ504710.2; AY687344.1; AY687343.1; AY687342.1; AJ504712.2; AJ504711.2; KF992212.1; KF992209.1; JN701804.1; SMSF01000006.1; QWFW01031295.1; LWLT01000006.1; JAIWQT010000026.1; JACWUT010000006.1;	insTGTACAATGCCATTAATATATTGTACAA
		Capra aegagrus	JXYW01067820.1; CBYH010035613.1; AJPT02026392.1;	
		Capra falconeri	JAWPPH010101386.1	
		Capra ibex	SJYO01041852.1	
		Capra sibirica	NIYN02007310.1	
	Ovis	*Ovis aries*	JN701803.1; JN560175.1; AJ784891.1; AY534901.1; AJ492562.1; KT283274.1; KT283273.1; KT283272.1; KT283270.1; KT283269.1; KT283268.1; KT283267.1; KT283266.1; KT283265.1; KT283264.1; KT283263.1; KT283262.1; KT283261.1; KT283260.1; KT283259.1; KT283258.1; KT283257.1; KT283256.1; KT283255.1; ACIV010650360.1; AMGL02018602.1; CBYI010094575.1; JAAFGP010000006.1; JACSDQ010000006.1; JAEMGP010000006.1; JAEVFA010000137.1; JAGTAQ010000006.1; JAGTXJ010000043.1; JAHUUQ010000411.1; JAHUUR010000810.1; JAJSZZ010000179.1; JAJTAA010000158.1; JAJTAB010000446.1; JAJTAC010001164.1; JAJTAD010000031.1; JAJTAE010000140.1; JAJTAF010000009.1; JAJTAG010000077.1; JAJTAH010000108.1; JAJTAI010000001.1; JAJTAJ010000087.1; JAJTAK010000097.1; JAJTAL010000208.1; JAJTAM010000338.1; JAJTAN010000028.1; JAJTAO010000125.1; JAJTAP010000614.1; JAJTAQ010000706.1; JAJTAR010000025.1; JAJTAS010000778.1; JAJTAT010000007.1; JAJTAU010000086.1; JAJTAV010000101.1; JAJTAW010000052.1; JAKFGC010000037.1; JAKFGD010000057.1; JAKJQE010000023.1; JAKJQF010000023.1; JAKJQG010000023.1; JAKJQH010000023.1; JAKJQI010000023.1; JAKJQJ010000023.1; JAKJQK010000023.1; JAKJQL010000023.1; JAKJQM010000637.1; JAKJQN010000023.1; JAKJQO010000023.1; JAKJQP010000023.1; JAKZEL010000006.1; JALAIX010000005.1; JAMFTI010000172.1; JAMFTJ010000486.1; JAMFTK010000023.1; JAMHGC010000249.1; JAMHGD010000007.1; JAMHGE010000023.1; JAVYAH010303531.1; JAWMPZ010000006.1; PEKD01002087.1	**delTGTACAA**
		Ovis ammon	NIWH01032118.1; SJYP01000021.1	
		Ovis ammon x Ovis aries	JALAIW010000004.1	
	Bubalus	*Bubalus bubalis*	KJ635888.1; KC951930.1; GU593719.1; AF529305.2; JN228894.1; ACZF03011528.1; AWWX01570220.1; LPUW01017969.1; NPZD01074909.1; PZYV01000022.1; VDCB01000012.1; VDCC01000007.1	delTGTACAATGCCATTAATATA
		Bubalus depressicornis	JAMXBS010096084.1	
		Bubalus kerabau	JARFXY010000007.1	
	Bos	*Bos taurus*	X59856.2; AF435922.1; CAXHSW010004588.1; CAXHSS010001722.1; CAXHSU010000334.1; AAFC05011015.1; CAJZAZ010000049.1; CAWUBE010000006.1; CAXHSO010004348.1; CAXHSP010001289.1; CAXHSQ010000953.1; CAXHSR010005963.1; CAXHST010007339.1; DAAA02018006.1; JAJQWI010000006.1; JAJQWL010000006.1; JARDUZ020000006.1;	
		Bos indicus	JAUBKJ010000009.1; JAPFII010000078.1; JAPFIJ010000064.1; JAKQXV010000026.1; JAKQXR010000026.1; JAKQXP010000026.1; JAKQXO010000026.1; JAJUAV010000026.1; JAJUAT010000026.1; JAJUAS010000026.1; JAJUAP010000026.1; JAJUAM010000026.1; JAJUAL010000026.1; JAJUAG010000026.1; JAJUAE010000026.1;	
		Bos grunniens	JANCMS010001299.1; VBZB01000005.1;	
		Bos frontalis	JAFDUV010757737.1	
		Bos grunniens x Bos taurus	VLPI01000482.1; VLPJ01000340.1	
		Bos indicus x Bos taurus	PUFT02000006.1; PUFS02000006.1; JAAIXW010000007.1; JAAIXV010000007.1; JAAIXU010000031.1; JAAIXT010000031.1; JAAIXS010002926.1; JAAIXR010000031.1	
		Bos mutus	VBQZ03000078.1; JANCMR010002070.1; AGSK01123304.1;	
		Bos gaurus	JACAOC010000006.1	
		Bos javanicus	JAVLEU010000006.1	
