# Supplementary material for: Identification and Validation of Genus/Species-Specific Short InDels in Dairy Ruminants
Source: BMC Vet Res. 2025 Mar 28;21:215. doi: 10.1186/s12917-025-04694-z (PMC11951546; doi:10.1186/s12917-025-04694-z)
Supplement: Supplementary file 5 — Additional file 5: Table 4 In silico species-specific InDel identification at the CSN1S2 locus in the genera Capra, Ovis, Bubalus, and Bos. The specific deletion for the genus Ovis is highlighted in gray. [file 12917_2025_4694_MOESM5_ESM.pdf]

Additional file 5 - Table 4 *In silico* species-specific InDel identification at *CSN1S2 locus* in *genus Capra*, *Ovis*, *Bubalus*, and *Bos*. The specific deletion for the *genus Ovis* is highlighted in grey.

Gene	Genus	Species/ hybrid	GeneBank accession number	InDel
CSN1S2	*Capra*	*Capra hircus*	JAIWQT010000026.1; JACWUT010000006.1; NIYN02007310.1; AJPT02026392.1; LWLT01000006.1; QWFW01031295.1; SMSF01000006.1	insAGAAATCAAATCTT
		Capra aegagrus	CBYH010035617.1; JXYW01067838.1	
		Capra falconeri	JAWPPH010024540.1	
		Capra ibex	SJYO01041852.1	
		Capra sibirica	NIYN02007310.1	
	Ovis	*Ovis aries*	KT283343.1; KT283344.1; KT283345.1; KT283350.1; KT283351.1; KT283352.1; KT283340.1; KT283341.1; KT283342.1; KT283346.1; KT283347.1; KT283348.1; KT283349.1; KT283353.1; KT283354.1; AMGL02018604.1; JAEVFA010000137.1; JAHUUQ010000411.1; JAHUUR010000810.1; JAJTAE010000140.1; JAJTAK010000097.1; JAJTAQ010000706.1; JAJTAW010000052.1; JAKFGC010000037.1; JAKJQG010000023.1; JAKJQO010000023.1; JAWMPZ010000006.1; PEKD01002087.1; CBYI010094582.1; JAAFGP010000006.1; JAEMGP010000006.1; JAGTAQ010000006.1; JAGTXJ010000043.1; JAJSZZ010000179.1; JAJTAA010000158.1; JAJTAB010000446.1; JAJTAC010001164.1; JAJTAD010000031.1; JAJTAF010000009.1; JAJTAG010000077.1; JAJTAH010000108.1; JAJTAI010000001.1; JAJTAJ010000087.1; JAJTAL010000208.1; JAJTAM010000338.1; JAJTAN010000028.1; JAJTAO010000125.1; JAJTAP010000614.1; JAJTAR010000025.1; JAJTAS010000778.1; JAJTAT010000007.1; JAJTAU010000086.1; JAJTAV010000101.1; JAKFGD010000057.1; JAKJQE010000023.1; JAKJQF010000023.1; JAKJQH010000023.1; JAKJQI010000023.1; JAKJQJ010000023.1; JAKJQK010000023.1; JAKJQL010000023.1; JAKJQM010000637.1; JAKJQN010000023.1; JAKJQP010000023.1; JAKZEL010000006.1; JAMFTI010000172.1; JAMFTJ010000486.1; JAMFTK010000023.1; JAMHGC010000249.1; JAMHGD010000007.1; JAMHGE010000023.1; JAVYAH010892373.1	delAGAAATCAAATCTT
		Ovis nivicola	CAFBRR010000237.1	
		Ovis canadensis	PVIS010023768.1	
		Ovis orientalis	JACSDQ010000006.1	
		Ovis ammon	NIWH01032118.1; SJYP01000021.1	
		Ovis ammon x Ovis aries	JALAIW010000004.1; JALAIX010000005.1	
	Bubalus	*Bubalus bubalis*	MW159136.1; MW159135.1; AWWX01524212.1; PZYV01000022.1; ACZF03000833.1; LPUW01338772.1; NPZD01074901.1; VDCB01000012.1; VDCC01000007.1	insAGAAATCAAAYCTT
		Bubalus depressicornis	JAMXBS010059713.1	
		Bubalus kerabau	JARFXY010000007.1	
	Bos	*Bos taurus*	M94327.1; CAXHSP010001289.1; CAXHSQ010000953.1; CAXHSS010006815.1; CAXHSU010000334.1; AAFC05011017.1; CAJZAZ010000049.1; CAWUBD010000006.1; CAWUBE010000006.1; CAWUBF010000007.1; CAXHSO010002397.1; CAXHSR010005963.1; CAXHST010007151.1; CAXHSW010004588.1; DAAA02018008.1; JAJQWI010000006.1; JAJQWL010000006.1; JAMBMV010000006.1; JANIWY010000005.1; JARDUZ020000006.1; JASJPV010001119.1; NKLS02000006.1; JAWKDW010000008.1	insAGAAATCAAATCTT
		Bos indicus	JAJUAD010000026.1; JAJUAE010000026.1; JAJUAF010000026.1; JAJUAH010000026.1; JAJUAI010000026.1; JAJUAJ010000026.1; JAJUAK010000026.1; JAJUAL010000026.1; JAJUAO010000026.1; JAJUAP010000026.1; JAJUAR010000026.1; JAJUAS010000026.1; JAJUAV010000026.1; JAJUAW010000026.1; JAKQXM010000026.1; JAKQXN010000026.1; JAKQXO010000026.1; JAKQXP010000026.1; JAKQXQ010000026.1; JAKQXS010000026.1; JAKQXT010000026.1; JAKQXU010000026.1; JAKQXV010000026.1; JAPFIJ010000064.1; JASFDU010570032.1; JAUBKJ010000009.1; AGFL01059864.1; JAJUAG010000026.1; JAJUAM010000026.1; JAJUAN010000026.1; JAJUAT010000026.1; JAKQXR010000026.1; JAPFII010000078.1; PRDE01000026.1; JAJUAQ010000026.1	
		Bos grunniens	JANCMS010001299.1; VBZB01000005.1	
		Bos frontalis	JAFDUV010372417.1; RBVW01000082.1	
		Bos grunniens x Bos taurus	VLPJ01000340.1; VLPI01000482.1	
		Bos indicus x Bos taurus	JAAIXS010002926.1; JAAIXT010000031.1; JAAIXV010000007.1; PUFT02000006.1; JAAIXR010000031.1; JAAIXU010000031.1; JAAIXW010000007.1; PUFS02000006.1;	
		Bos mutus	AGSK01123296.1; JANCMR010002070.1; VBQZ03000078.1	
		Bos gaurus	JACAOC010000006.1	
		Bos javanicus	JAVLEU010000006.1	
