# Supplementary material for: Identification and Validation of Genus/Species-Specific Short InDels in Dairy Ruminants
Source: BMC Vet Res. 2025 Mar 28;21:215. doi: 10.1186/s12917-025-04694-z (PMC11951546; doi:10.1186/s12917-025-04694-z)
Supplement: Supplementary file 6 — Additional file 6: Fig. 2 Comparison of CSN1S2 partial intron 1 nucleotide sequences of representative species belonging to the Artiodactyla and Perissodactyla orders. The dashes represent nucleotides identical to those in the upper lines. The short InDels are highlighted in gray. 1: Ovis aries (GenBank KT283354.1 from 610 to 696); 2: Ovis aries (GenBank JAWMPZ010000006.1 from 91390711 to 91390797); 3: Capra hircus (GenBank LWLT01000006.1 from 86078347 to 86078447); 4: Bubalus bubalis (Mediterranean GenBank MW159135.1 from 2404 to 2504, and Murrah GenBank VDCC01000007.1 from 32346792 to 32346892); 5: Bubalus bubalis (Mediterranean breed GenBank MW159136.1 from 2393 to 2493); 6: Bubalus bubalis (Kerabau swamp GenBank JARFXY010000007.1 from 89744003 to 89744103, and Depressicornis GenBank JAMXBS010059713.1 from 4606 to 4706); 7: Bos taurus (GenBank M94327.1 from 3584 to 3684); 8: Bos indicus (GenBank PRDE01000026.1 from 87254016 to 87254116); 9: Bos grunniens (GenBank VBZB01000005.1 from 35840127 to 35840227); 10: Rangifer tarandus (GenBank OX596114.1 from 19874491 to 19874593), and Cervus elaphus (GenBank OU343083.1 from 30339433 to 30339535, complement); 11: Muntiacus reevesi (GenBank OZ005646.1 from 30071163 to 30071265); 12: Sus scrofa (GenBank LUXU01069711.1 from 564154 to 564256); 13: Equus caballus (GenBank JAPJZS010003086.1 from 51148228 to 51148330), Equus asinus (GenBank JREZ01000259.1 from 17762 to 17864), and Equus quagga (GenBank JAKJSB010001568.1 from 100903916 to 100904018, complement); 14: Ceratotherium simum (GenBank AKZM01002854.1 from 54353 to 54455); 15: Tapirus indicus (GenBank JAVSPQ010000004.1 from 54488574 to 54488676). [file 12917_2025_4694_MOESM6_ESM.pdf]

```
                                                                                                                         SUBORDERS       ORDERS

TCATAGTATTAGAGATTGAGCTGGAGGGGATCTT              CTTAGTTAAAAATCTTGTTTGGTGGTTATTCT  GTCAGGTTCAGTGATGTTCAG 1
----------------------------------              ---------------C----------------  --------------------- 2
----------------------------------AGAAATCAAATCTT---------------CA---------------  ------------A-G------ 3
----------------------------------AGAAATCAAATCTT-----------G-------------------C  --------------------- 4
----------------------------------AGAAATCAAACCTT--------C--G-------------------C  --------------------- 5
----------------------------------AGAAATCAAATCTT-----------G-------------------C  --------------------- 6   Ruminantia      Artiodactyla
----------------------------------AGAAATCAAATCTT---------------CA---------------  --------------------- 7
----------------------------------AGAAATCAAATCTT---------------CR---------------  --------------------- 8
----------------------------------AGAAATCAAATCTT---------------C----------------  --------------------- 9
--------------------A-------------AGAAATCAAATCTT---------------CA--------------AA---------------------- 10
--------------------A-------------ATAAATCAAATCTT---------------CA--------------AA---------------------- 11
---C---------------------A--------GGAAATCAAATCTCA---------TC---CT----T-A-A-----AAAA-G-TA-----------C--T 12  Suiformes
---C--C-C---------G------A--------AGAAATCAAGGATC---CA-C--GTGC--CT-A--C-A-A----TGAAA-G-A-GA-----G------- 13  Hippomorpha
---C--------------------TA--------AGAAATCAAGTCTC---T--C--GTCC--CT----C-A-A----TGAAC-G-A-GG------------- 14  Ceratomorpha    Perissodactyla
--GC--G--------C---------A--------AGAAATTGAGTCTC---C--C--GTCC--GT----C-A-A----TGAAA-G---GG-------A----C 15
```
