# Supplementary material for: Identification and Validation of Genus/Species-Specific Short InDels in Dairy Ruminants
Source: BMC Vet Res. 2025 Mar 28;21:215. doi: 10.1186/s12917-025-04694-z (PMC11951546; doi:10.1186/s12917-025-04694-z)
Supplement: Supplementary file 7 — Additional file 7: Table 5 In silico species-specific InDel identification at the MSTN locus in the genera Capra, Ovis, Bubalus, and Bos. The specific deletion for Bos taurus is highlighted in gray. [file 12917_2025_4694_MOESM7_ESM.pdf]

Gene	Genus	Species/ hybrid	GeneBank Accession	InDel
MSTN	*Capra*	*Capra hircus*	JX078969.1; JX078968.1; JN012228.1; EF591039.1; EF588035.1; EF588033.1; EF588032.1; EF588031.1; EF588024.1; EF588017.1; DQ167575.2; AF393619.1; AJPT02030578.1; JACWUT010000002.1; JAIWQT010000022.1; LWLT01000002.1; QWFW01048360.1; SMSF01000002.1	insGAGTAGGTTATGGCTT
		Capra aegagrus	CBYH010040833.1; JXYW01015122.1	
		Capra falconeri	JAWPPH010102605.1	
		Capra sibirica	NIYN02012871.1	
	Ovis	*Ovis aries*	MH025940.1; MH025933.1; JN856476.1; JN856475.1; JN856474.1; JN856473.1; DQ990914.1; DQ530260.1; AF393618.1; CBYI010111740.1; ACIV010214208.1; AMGL02007816.1; JAVYAH010484028.1; JAWMPZ010000002.1; PEKD01000915.1; JAMHGE010000012.1; JAMHGD010000119.1; JAMHGC010000089.1; JAMFTK010000012.1; JAMFTJ010000386.1; JAMFTI010000003.1; JAKJQP010000012.1; JAKJQO010000012.1; JAKJQN010000012.1; JAKJQM010000051.1; JAKJQL010000012.1; JAKJQK010000012.1; JAKJQJ010000012.1; JAKJQI010000012.1; JAKJQH010000012.1; JAKJQG010000012.1; JAKJQF010000012.1; JAKJQE010000012.1; JAKFGD010000003.1; JAKFGC010000030.1; JAJTAW010000027.1; JAJTAV010000169.1; JAJTAU010000093.1; JAJTAT010000003.1; JAJTAS010000059.1; JAJTAR010000077.1; JAJTAQ010002292.1; JAJTAP010001953.1; JAJTAO010000010.1; JAJTAN010000111.1; JAJTAM010000003.1; JAJTAL010000166.1; JAJTAK010000062.1; JAJTAJ010000031.1; JAJTAI010000039.1; JAJTAH010000045.1; JAJTAG010000205.1; JAJTAF010000022.1; JAJTAE010001625.1; JAJTAE010000035.1; JAJTAD010000058.1; JAJTAC010000403.1; JAJTAB010000366.1; JAJTAA010000012.1; JAJSZZ010000019.1; JAHUUR010000589.1; JAHUUQ010000103.1; JAGTXJ010000011.1; JAGTAQ010000002.1; JAEVFA010000126.1; JAEMGP010000002.1; JACSDQ010000002.1; JAAFGP010000002.1.	
		Ovis ammon	JAKZEL010000004.1; SJYP01000279.1	
		Ovis ammon x Ovis aries	JALAIX010000003.1; JALAIW010000002.1	
		Ovis canadensis	PVIS010026292.1;	
		Ovis nivicola	CAFBRR010000952.1	
	Bubalus	*Bubalus bubalis*	DQ091762.1; LPUW01020148.1; ACZF03036821.1; AWWX01583076.1; NPZD01009662.1; PZYV01000012.1; VDCB01000015.1; VDCC01000002.1;	
		Bubalus depressicornis	JAMXBS010027670.1	
		Bubalus kerabau	JARFXY010000003.1	
	Bos	*Bos taurus*	MK214682.1; AF320998.1; AB076403.1; AY850105.1; JQ711180.1; CAXHSP010000184.1; CAXHSO010002684.1; CAXHSW010007115.1; CAXHSQ010003235.1; CAXHSS010002069.1; AAFC05004680.1; CAJZAZ010000002.1; CAWUBD010000003.1; CAWUBE010000002.1; CAWUBF010000002.1; CAXHST010006835.1; CAXHSU010005115.1; CAXHSV010009125.1; DAAA02003905.1; JAJQWI010000002.1; JAJQWL010000002.1; JAMBMV010000002.1; JANIWY010000001.1; JARDUZ020000002.1; JASJPV010000704.1; NKLS02000002.1; CM078292.1	delGAGTAGGTTATGGCTT
		Bos gaurus x Bos taurus (Gaur x Piedmontese trio)	OX258956.1	
		Bos indicus breed Nelore	AGFL01013059.1	
		Bos indicus breed Gir	PRDE01000012.1	
		Bos indicus x Bos taurus breed Angus x Brahman	PUFT02000002.1; JAAIXR010000034.1; JAAIXU010000034.1; JAAIXW010000006.1	delGAGTAGGTTATGGCTT
			PUFS02000002.1; JAAIXV010000006.1; JAAIXT010000034.1; JAAIXS010003096.1	insGAGTAGGTTATGGCTT
		Bos indicus breed Guanling: BioSample: SAMN23846328	JAKQXU010000012.1; JAJUAH010000012.1	delGAGTAGGTTATGGCTT
			JAJUAI010000012.1	insGAGTAGGTTATGGCTT
		Bos indicus breed Lincanggaofeng: BioSample: SAMN23846331	JAJUAM010000012.1	delGAGTAGGTTATGGCTT
			JAKQXR010000012.1; JAJUAL010000012.1	insGAGTAGGTTATGGCTT
		Bos indicus breed Weizhou: BioSample: SAMN23846332	JAKQXQ010000012.1; JAJUAN010000012.1	delGAGTAGGTTATGGCTT
			JAJUAO010000012.1; JAJUAT010000012.1	insGAGTAGGTTATGGCTT
		Bos indicus breed	JAKQXM010000012.1	delGAGTAGGTTATGGCTT

		Weining: BioSample: SAMN23846336	JAJUAU010000012.1	insGAGTAGGTTATGGCTT
		Bos grunniens x Bos taurus: BioSample: SAMN12153487	VLPJ01000219.1	delGAGTAGGTTATGGCTT
			VLPI01000022.1	insGAGTAGGTTATGGCTT
		Bos indicus	AY794986.1; JAJUAD010000012.1; JAJUAE010000012.1; JAJUAG010000012.1; JAJUAJ010000012.1; JAJUAK010000012.1; JAJUAO010000012.1; JAJUAP010000012.1; JAJUAQ010000012.1; JAJUAS010000012.1; JAJUAU010000012.1; JAJUAV010000012.1; JAJUAW010000012.1; JAKQXN010000012.1; JAKQXO010000012.1; JAKQXP010000012.1; JAKQXS010000012.1; JAKQXT010000012.1; JAKQXV010000012.1; JAMBMU010000002.1; JAPFII010000022.1; JAPFIJ010000022.1; JASFDU010183844.1; JAUBKJ010000074.1	insGAGTAGGTTATGGCTT
		Bos grunniens	JN642607.1; EU926670.1; JANCMS010001307.1; VBZB01000002.1	
		Bos mutus	VBQZ03000004.1; AGSK01165038.1; JANCMR010002063.1	
		Bos gaurus	JACAOC010000017.1	
		Bos frontalis	VBQZ03000004.1; JAFDUV010761506.1; RBVW01003794.1	
		Bos javanicus	JAVLEU010000002.1	
		Bos taurus breed Yunling cattle	JAWKDW010000003.1	

Additional file 7 - Table 5. *In silico* species-specific InDel identification at *MSTN locus* in *genus Capra*, *Ovis*, *Bubalus*, and *Bos*. The specific deletion for *Bos taurus* is highlighted in grey.
