# Supplementary material for: Identification and Validation of Genus/Species-Specific Short InDels in Dairy Ruminants
Source: BMC Vet Res. 2025 Mar 28;21:215. doi: 10.1186/s12917-025-04694-z (PMC11951546; doi:10.1186/s12917-025-04694-z)
Supplement: Supplementary file 8 — Additional file 8: Fig. 3 Comparison of the partial MSTN intron 1 nucleotide sequences of representative species belonging to the Artiodactyla and Perissodactyla orders. The dashes represent nucleotides identical to those in the upper lines. The short InDels are highlighted in gray. 1: Bos taurus (GenBank AB076403.1 from 1159 to 1262), and Bos indicus x Bos taurus hybrids (GenBank PUFT02000002.1 from 127935602 to 127935705); 2: Bos grunniens (GenBank JN642607.1 from 1097 to 1216), Bos mutus (GenBank VBQZ03000004.1 from 47972658 to 47972777), Bos frontalis (GenBank RBVW01003794.1 from 79189 to 79308), Bos javanicus (GenBank JAVLEU010000002.1 from 6312509 to 6312628), Bos gaurus (GenBank JACAOC010000017.1 from 1124918 to 1125037), Bos indicus (GenBank AY794986.1 from 1159 to 1278), Bos indicus x Bos taurus hybrids (GenBank PUFS02000002.1 from 6036628 to 6036747), Bison bison (GenBank JPYT01086742.1 from 5189 to 5308), and Bos taurus breed Yunling (GenBank JAWKDW010000003.1 from 131646859 to 131646740, complement); 3: Bubalus kerabau breed swamp (GenBank JARFXY010000003.1 from 59857473 to 59857592), and Bubalus depressicornis (GenBank JAMXBS010027670.1 from 21336 to 21455); 4: Bubalus bubalis breed Mediterranean (GenBank DQ091762.1 from 1065 to 1184), and Bubalus bubalis breed Murrah (GenBank VDCC01000002.1 from 58035442 to 58035561); 5: Rangifer tarandus (GenBank OX596096.1 from 68947317 to 68947436), and Cervus elaphus (GenBank OU343110.1 from 10575652 to 10575771); 6: Ovis aries (GenBank MH025940.1 from 2210 to 2329), and Capra hircus (GenBank JX078969.1 from 1224 to 1343); 7: Camelus dromedarius (GeneBan LSZX01094446.1 from 13519 to 13638), Camelus ferus (GeneBanVSZR01000033.1 from 59262525 to 59262644), and Camelus bactrianus (GenBank CAOW010387223.1 from 1829 to 1948); 8: Lama glama (GeneBan PNXU01000637.1 from 236134 to 236253), and Vicugna pacos (GenBank JEMW01017296.1 from 84546 to 84665); 9: Sus scrofa (GenBank EF490990.1 from 2235 to 2353); 10: Equus caballu [file 12917_2025_4694_MOESM8_ESM.pdf]

```
                                                                                                                                        SUBORDERS        ORDERS

AAATCATGAGCTAATCAGCAGAAAATTC TAAGAAATAAACATTTTAATT                ACAAAGTTCCACTTATACCCTGACCATGGTACTATTGTTGAGAGTACCTGGTCTG 1
---------------------------- ---------------------GAGTAGGTTATGGCTT------------------------------------------------------- 2
--------------------------G- ---------------------GAGTAGGTTATGGCTT------------------------------------------------------- 3   Ruminantia
--------------------------G- -------------C-------GAGTAGGTTATGGCTT------------------------------------------------------- 4
---------------T----------G- -------------C-------GAGTAGGTTATGGCTT------------------------------------------------------- 5                  Artiodactyla
-----------C---G----------G- ---------------------GAGTAGGTTATGGCTT-------C----------------------------------------------- 6
---------------G-TT-------G- C-------------------CAAATAGGTTATGGCTC-------C-T---------T--G-------G------------A----C------ 7   Tylopoda
-------------G-G-TT-------G- C-------------------CAAATAGGTTATGGCTC------CC-T---------T--G-------G------------A----C------ 8
T---T----------T-T--------G- C----------------- -CAAGTAGGTTATGGCTC-------C-TG--------T----------------------------CT----- 9   Suiformes
T--------------T-T--T-----G-C---T----------------CAAAGAGGTTATAGCTC  -G---C-TG----C---T-------A----------------C---C--GG-- 10
T--G-------A-    T--T-----G-C---T----------------CAAAGAGGTTATAGCTC  -G---C-TG----C---T-------A----------------C---C--GG-- 11  Hippomorpha
T----------A-    T--T-----G-C---T----------------CAAAGAGGTTATAGCTC  -G---C-TG----C---T-------A----------------C---C--GG-- 12                 Perissodactyla
T--------------T-T--------G-C-------C-------A----CCAGTAGGTTATGGCTC---G---C-T-----C---T---A------T---C------A-G----C------ 13  Ceratomorpha
T--------------T-T--------G-T--------------------CACATAGGTTATGGTTC---G---C-A---------T--------------------G-------------- 14
```
