# Supplementary material for: Identification and Validation of Genus/Species-Specific Short InDels in Dairy Ruminants
Source: BMC Vet Res. 2025 Mar 28;21:215. doi: 10.1186/s12917-025-04694-z (PMC11951546; doi:10.1186/s12917-025-04694-z)
Supplement: Supplementary file 9 — Additional file 9: Fig. 4 Comparison of the PRLR sequences of partial exon 10 with the homologous sequences available in the database for the main species belonging to the Artiodactyla and Perissodactyla orders. The dashes represent nucleotides identical to those in the upper lines. The short Indels are highlighted in gray. 1: Bubalus bubalis (Mediterranean, GenBank MF461277.1 from 12141 to 12193, Murrah GenBank XM_025270632.3 from 2207 to 2259, Kerabau swamp, GenBank JARFXY010000018.1 from 40954223 to 40954275, Depressicornis, GenBank JAMXBS010060037.1 from 50052 to 50104, and Syncerus caffer, GenBank SJXX02054938.1 from 1119837 to 1119889, complement); 2: Bos taurus (GenBank OY997248.1 from 42476450 to 42476509), Bos indicus (GenBank XM_019983032.1 from 2188 to 2247), Bos javanicus (GenBank XM_061393756.1 from 2862 to 2921), Bos mutus (GenBank XM_005907371.2 from 1947 to 2006), and Bison bison (GenBank XM_010853807.1 from 2122 to 2181); 3: Tragelaphus oryx (GenBank SJYK013989325.1 from 1715 to 1774, complement); 4: Capra hircus (GenBank XM_061393756.1 from 2759 to 2818), and Ovis aries (GenBank KC734660.1 from 503 to 562); 5: Cervus elaphus (GenBank XM_043887317.1 from 2217 to 2278), and Rangifer tarandus (GenBank OX459950.2 from 41714039 to 41714100); 6: Camelus dromedarius (GenBank XM_031443888.1 from 2286 to 2349), Camelus ferus (GenBank XM_006194514.3 from 2238 to 2301), and Camelus bactrianus (GenBank XM_010955538.2 from 2224 to 2287); 7: Vicugna pacos (GenBank XM_006199659.3 from 2235 to 2298); 8: Lama glama (GenBank DQ206831 from 1068 to 1127); 9: Sus scrofa (GenBank HM059030.1 from 1105 to 1169); 10: Equus asinus (GenBank XM_014831229.2 from 2492 to 2549), and Equus quagga (GenBank XM_046671632.1 from 2414 to 2477; 11: Equus caballus (GenBank XM_001500104.4 from 2757 to 2820); 12: Ceratotherium simum simum (GenBank XM_014781582.1 from 2216 to 2279); 13: Tapirus indicus (GenBank JAVSPQ010000006.1 from 118655899 to 118655962). [file 12917_2025_4694_MOESM9_ESM.pdf]

```
                                                                          SUBORDERS        ORDERS

CTCAAGAACAGGGTTAGAATGA        CAACTCCCAGTTCACTC   TTCATTCTCT  ATTT 1
----------------------CACTACC -----------------   --A-------  ---- 2
-------------------C--CACTACC -----------------   ----------  ---- 3    Ruminantia
------------A---------CACTACC -----------------   ----------  ---- 4
-------G--------------CACTACC -----------------   ----------GT---- 5                     Artiodactyla
---C--C-TG---------G--CACTACCAA-----AG-A-------CTT----CG---C  ---- 6
---C--C-TG---------G--CACTACCAA-----AG---------CTT----CG---C  ---- 7    Tylopoda
---C--C-TG--------- --CACTACCA -----AG-----C---  T-----G---C  ---- 8
---G-A--TG------------CACTACCCA-----A----------CTC-----G---C  ---- 9    Suiformes
-G------GCT-----------CACTACTAA-----A--A---G---CTT-----G---C  ---- 10   Hippomorpha
-G-G----GCT-T---------CACTACTAA-----A--A---G---CTT-----G---C  ---- 11                    Perissodactyla
--------TGT-----------CACTACTAA-----A----------CTC-----G---C  ---- 12   Ceratomorpha
---G----GGT----G---C--CACTACTAA-----A----------CTC--TG-G---C ---- 13
```
