# Supplementary material for: Identification and Validation of Genus/Species-Specific Short InDels in Dairy Ruminants
Source: BMC Vet Res. 2025 Mar 28;21:215. doi: 10.1186/s12917-025-04694-z (PMC11951546; doi:10.1186/s12917-025-04694-z)
Supplement: Supplementary file 10 — Additional file 10: Table 6: In silico species-specific InDel identification at the PRLR locus in the genera Capra, Ovis, Bubalus, and Bos. The specific deletion for the genus Bubalus is highlighted in gray. [file 12917_2025_4694_MOESM10_ESM.pdf]

Additional file 10 - Table 6. *In silico* specie-specific InDel identification at *PRLR locus* in *genus Capra*, *Ovis*, *Bubalus*, and *Bos*. The specific deletion for the *genus Bubalus* is highlighted in grey.

Gene	Genus	Species/ hybrid	GeneBank accession number	InDel
PRLR	*Capra*	*Capra hircus*	CP162692.1; CP162722.1; CP155501.1; CP162228.1; XM_018065467.1; XM_013972806.2; XM_018065466.1; XM_013972804.2; XM_013972803.2; ON258666.1; ON258665.1; OM418864.1; OM418863.1; KJ572972.1; C109741.1; MT897488.1; MT897486.1; MT897485.1; MT897484.1; MT897483.1; MT897487.1; LWLT01000017.1; JACWUT010000020.1; AJPT02020844.1; JBBJUA010000022.1; JAIWQT010000012.1; QWFW01025655.1; SMSF01000020.1; QWFW01018215.1	insCACTACC
		Capra aegagrus	JXYW01183532.1; CBYH010037235.1	
		Capra falconeri	JAWPPH010015354.1	
		Capra sibirica	NIYN02009600.1	
	Ovis	*Ovis aries*	CP162722.1; CP162288.1; CP162352.1; CP162255.1; KC734660.1; KC734656.1; KC734647.1; KC734653.1; KC734652.1; KC734651.1; KC734648.1; KC734649.1; AF041979.1; PEKD01003415.1; JBEJUG010000008.1; JAWMPZ010000016.1; JAMFTJ010000513.1; JAKJQP010000008.1; JAKJQM010000888.1; JAKJQF010000008.1; JAKJQE010000008.1; JAKFGD010000023.1; JAKFGC010000062.1; JAJTAT010000005.1; JAJTAQ010000539.1; JAJTAP010000497.1; JAJTAO010000099.1; JAJTAJ010001470.1; JAJTAH010000063.1; JAJTAC010000161.1; JAHUUR010000555.1;JAHUUQ010000060.1; JAGTAQ010000016.1; JAEVFA010000122.1; JAMFTK010000008.1; JAMFTI010000250.1; JAVYAH010732814.1; JAMHGE010000008.1; JAMHGD010000006.1; JAMHGC010000004.1; AKJQO010000008.1; JAKJQN010000008.1; JAKJQK010000008.1; JAKJQJ010000008.1; JAKJQI010000008.1; JAJTAV010000086.1; JAJTAS010000038.1; JAJTAN010000013.1; JAJTAI010000108.1; JAJTAF010000039.1; JAJTAE010000002.1; JAJTAD010000286.1; JAJTAB010000085.1; JAJTAA010000024.1;JAGTXJ010000077.1; JAEMGP010000016.1; JAAFGP010000016.1; CBYI010081595.1; ACIV011696388.1; JAKJQH010000008.1; JAKJQG010000008.1; JAJTAM010000008.1; JAJTAK010000058.1; JAJTAL010000432.1; JAJTAG010000135.1; AMGL02003332.1; JBDLWQ010000016.1; JAJTAU010000021.1; JAJTAR010000291.1; JAKJQL010000008.1; JAJSZZ010000144.1	insCACTACC
		Ovis nivicola	CAFBRR010000034.1;	
		Ovis canadensis	CP011901.1; PVIS010000630.1; JBGCUV010000016.1;	
		Ovis ammon	SJYP01000071.1; NIWH01032642.1; JAKZEL010000017.1; SJYP01000071.1; NIWH01032642.1; JAKZEL010000017.1;	
		Ovis ammon x Ovis aries	JALAIX010000015.1	
	Bubalus	*Bubalus bubalis*	MF461277.1; XM_025270634.3; XM_025270635.3; XM_025270633.3; XM_025270632.3; GQ339914.1; VDCC01000019.1; VDCB01000010.1; PZYV01000011.1; NPZD01228838.1;LPUW01085823.1; WWX01527174.1; ACZF03002371.1; AWWX01527174.1	delCACTACC
		Bubalus depressicornis	JAMXBS010060037.1	
		Bubalus kerabau	JARFXY010000018.1	
	Bos	*Bos taurus*	OY997248.1; OX344709.1; XM_024981211.2; XM_024981210.2; XM_024981208.2; XM_005221579.5; XM_024981203.2; XM_024981206.2; XM_024981207.2; XM_005221576.5; XM_005221577.5; XM_024981202.2; XM_005221575.5; LR962876.1; LR962751.1; MW234418.1; MW234417.1; MW234416.1; NM_001039726.2; NM_174155.3; AJ966356.4; AF027403.1; L02549.1; CAXHSQ010005991.1; NKLS02000020.1; JBEFCS010000020.1; JBEFCR010000020.1; JAWKDW010000019.1; JASJPV010000586.1; JARDUZ020000020.1; JANIWY010000017.1; JAMBMV010000020.1; JAJQWL010000020.1; JAJQWI010000020.1; DAAA02050661.1; CAXHSS010001515.1; CAXHSP010002923.1; CAXHSO010000183.1; CAWUBF010000019.1; CAWUBE010000020.1; CAWUBD010000019.1; CAJZAZ010000033.1; AAFC05029971.1; CAXHST010007939.1; CAXHSU010001030.1; CAXHSV010000535.1; CAXHSR010002888.1; CAXHSW010006238.1	insCACTACC
		Bos indicus	XM_019983032.1; XM_019983031.1; PRDE01000013.1; JAUBKJ010001012.1; JASFDU010039985.1; JAPFIJ010000009.1; JAPFII010000009.1; JAMBMU010000020.1; JAKQXV010000013.1; JAKQXU010000013.1; JAKQXT010000013.1; JAKQXS010000013.1; JAKQXR010000013.1; JAKQXQ010000013.1; JAKQXP010000013.1; JAKQXO010000013.1; JAKQXN010000013.1; JAKQXM010000013.1; JAJUAW010000013.1; JAJUAV010000013.1; JAJUAU010000013.1; JAJUAT010000013.1; JAJUAS010000013.1; JAJUAQ010000013.1; JAJUAP010000013.1; JAJUAO010000013.1; JAJUAN010000013.1; JAJUAM010000013.1; JAJUAL010000013.1; JAJUAK010000013.1; JAJUAJ010000013.1; JAJUAI010000013.1; JAJUAH010000013.1; JAJUAG010000013.1; JAJUAF010000013.1; JAJUAE010000013.1; JAJUAD010000013.1; AGFL01161847.1	
		Bos grunniens	VBZB01000024.1; VBQZ03000133.1; JANCMS010001311.1;	

		Bos frontalis	RBVW01000258.1; JAFDUV011072478.1;	
		Bos grunniens x Bos taurus	VLPJ01000010.1; VLPI01000010.1;	
		Bos indicus x Bos taurus	XM_027519928.1; XM_027519927.1; XM_027519925.1; XM_027519924.1; PUFT02000020.1; PUFS02000020.1; JAAIXW010000021.1; JAAIXV010000021.1; JAAIXU010000020.1; JAAIXT010000020.1; JAAIXS010002109.1; JAAIXR010000020.1	
		Bos mutus	XM_005907371.2; JANCMR010002053.1; AGSK01164430.1	
		Bos gaurus x Bos taurus	OX258974.1	
		Bos javanicus	XM_061393758.1; XM_061393756.1; XM_061393755.1; XM_061393754.1; XM_061393753.1; XM_061393752.1; XM_061393751.1; XM_061393750.1; JAVLEU010000020.1;	
		Bos gaurus	JACAOC010000020.1	
