# Supplementary material for: Identification and Validation of Genus/Species-Specific Short InDels in Dairy Ruminants
Source: BMC Vet Res. 2025 Mar 28;21:215. doi: 10.1186/s12917-025-04694-z (PMC11951546; doi:10.1186/s12917-025-04694-z)
Supplement: Supplementary file 11 — Additional file 11: Uncropped gels used for Figure 1, 2, 3, 4 and 5. [file 12917_2025_4694_MOESM11_ESM.pdf]

Figure and Legends

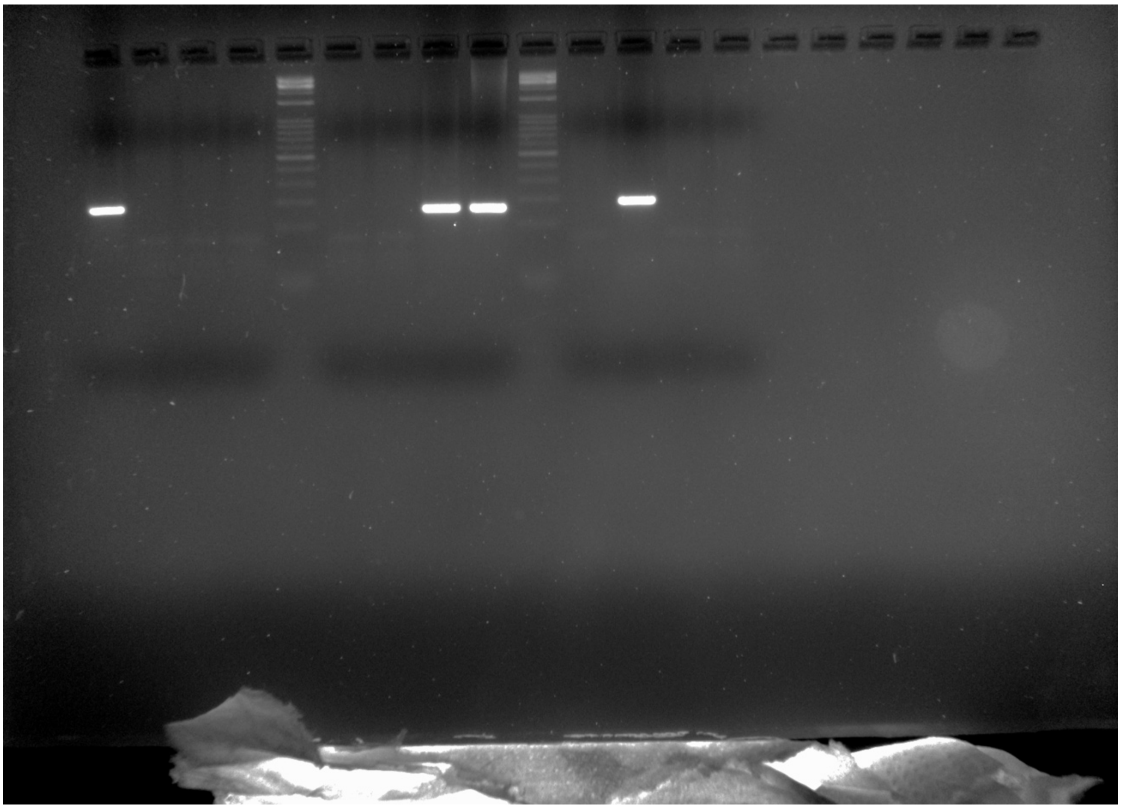

Fig. 1 Identification by AS-PCR of carriers of InDels at the *CSN1S1* 5'UTR: (**A**) 28 bp insertion (TGTACAATGCCATTAATATATTGTACAA) (primer reverse: CSN1S1ins28); (**B**) 20 bp deletion (TGTACAATGCCATTAATATA) (primer reverse: CSN1S1del20), and (**C**) 7 bp deletion (TGTACAA) (primer reverse: CSN1S1del7) in *Capra hircus* (**1**), *Ovis aries* (**2**), *Bos taurus* (**3**), and *Bubalus bubalis* (**4**). M=Marker 1 kb Opti-DNA Ladder (0.1–10 kb) (Biolabs)

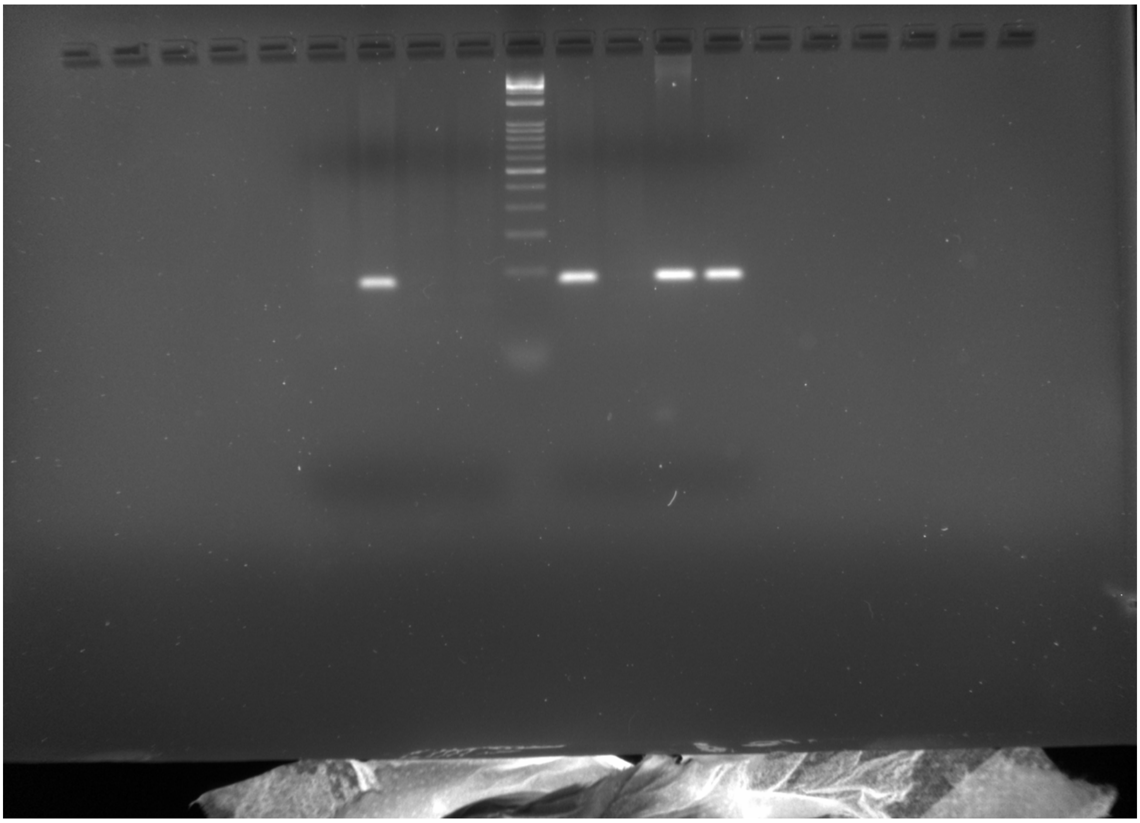

Fig. 2 Identification by AS-PCR of carriers of the 14 bp InDel (AGAAATCAAATCTT) at *CSN1S2* intron 1: (**A**) 14 bp deletion (primer reverse: CSN1S2del14); (**B**) 14 bp insertion (primer reverse: CSN1S2ins14) in *Capra hircus* (**1**), *Ovis aries* (**2**), *Bos taurus* (**3**), and *Bubalus bubalis* (**4**). M=Marker 1 kb Opti-DNA Ladder (0.1–10 kb) (Biolabs)

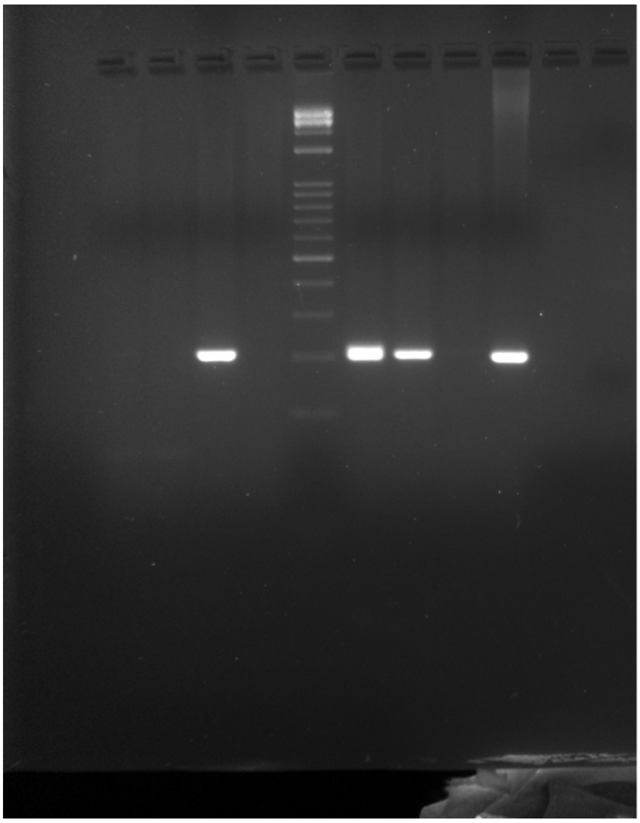

Fig. 3 Identification by AS-PCR of carriers of the 16 bp InDel (GAGTAGGTTATGGCTT) at *MSTN* intron 1: (**A**) 16 bp deletion (primer reverse: MSTNdel16); (**B**) 16 bp insertion (primer reverse: MSTNinsl16) in *Capra hircus* (**1**), *Ovis aries* (**2**), *Bos taurus* (**3**), and *Bubalus bubalis* (**4**). M=Marker 1 kb Opti-DNA Ladder (0.1–10 kb) (Biolabs)

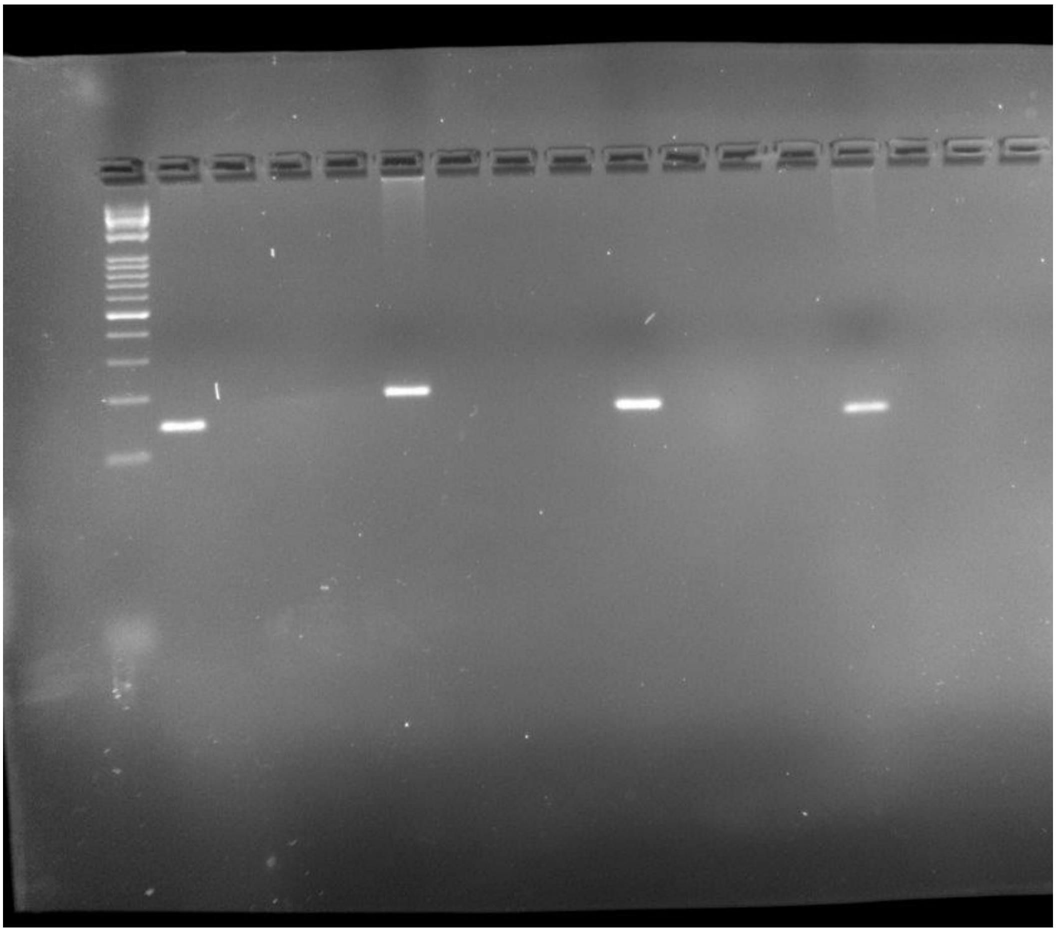

Fig. 4 Electrophoretic patterns of simplex species-specific amplification by using new (**A** and **D**) and previously designed (**B** and **D**) specific primer pairs. **A**) *PRLR*; **B**) *MSTN*; **C**) *CSN1S1*; **D**) *CSN1S2*. were from: *Mediterranean river buffalo* (patterns 1, 6, 10 and 14), *Bos taurus* (patterns 2, 5, 11 and 15), *Capra hircus* (patterns 3, 7, 9, 16), and *Ovis aries* (patterns 4, 8, 12 and 13). M: 1 kb Opti-DNA Marker, Applied Biological Materials

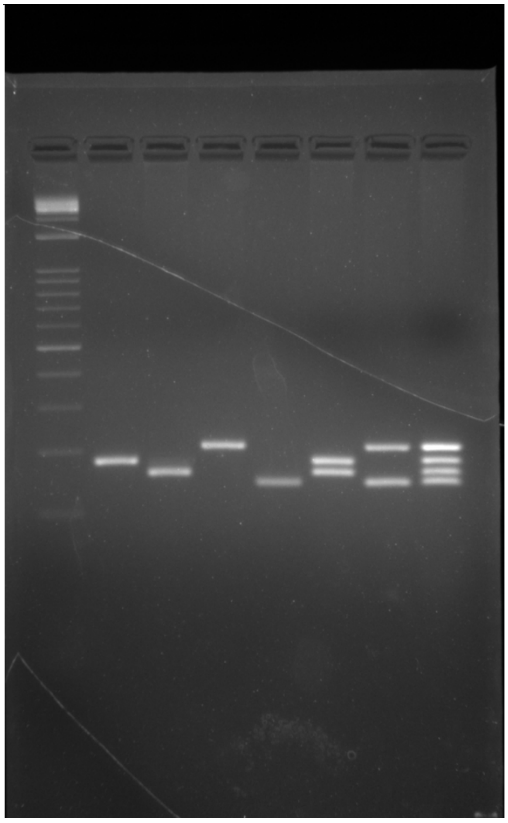

Fig. 5 Electrophoretic patterns of amplification products by TetraSS-PCR. Lane 1, *Capra hircus* (183 bp); line 2, *Ovis aries* (162 bp); line 3, *Bos taurus* (211 bp); line 4, Mediterranean river buffalo (144 bp). Lane 5, mixture of DNA from *Capra hircus* and *Ovis aries*; lane 6, mixture of DNA from *Mediterranean river buffalo* and *Bos taurus*; lane 7, DNA amplification products from *Mediterranean river buffalo*, *Bos taurus*, *Capra hircus*, and *Ovis aries*. M) 1 kb Opti-DNA Marker, Applied Biological Materials
